# High Melphalan Exposure Increases the Risk of Graft-Versus-Host Disease in Pediatric Patients Undergoing Alpha-Beta T-Cell Depleted Haploidentical Transplantation

**DOI:** 10.1016/j.jtct.2025.03.020

**Published:** 2025-04-02

**Authors:** Christopher C. Dvorak, Soohee Cho, Gabriel Salinas Cisneros, Christine S. Higham, Julia Chu, Lena E. Winestone, William C. Temple, Sandhya Kharbanda, Kristin A. Shimano, Serine Avagyan, Philip T. Pauerstein, James N. Huang, Geoffrey Cheng, Nahal Lalefar, Paibel Aguayo-Hiraldo, Ron J. Keizer, Michael A. Pulsipher, Janel R. Long-Boyle

**Affiliations:** 1 Division of Pediatric Allergy, Immunology, and Bone Marrow Transplantation, University of California San Francisco, San Francisco, California; 2 Division of Hematology and Oncology, Intermountain Primary Children’s Hospital, Huntsman Cancer Institute, Spencer Fox Eccles School of Medicine at The University of Utah, Salt Lake City, Utah; 3 Division of Pediatric Oncology, University of California San Francisco, San Francisco, California; 4 Division of Pediatric Hematology, University of California San Francisco, San Francisco, California; 5 Division of Pediatric Hematology, Oncology, and Transplantation and Cellular Therapy, University of Southern California, Children’s Hospital Los Angeles, Los Angeles, California; 6 Insight RX, San Francisco, California; 7 Department of Clinical Pharmacy, University of California San Francisco, San Francisco, California

**Keywords:** Melphalan, Pharmacokinetics, GVHD, Haploidentical

## Abstract

**Background:**

Melphalan is often used as the backbone agent for conditioning prior to A/B-T-cell depleted (A/B-TCD) hematopoietic cell transplant (HCT) due to lower rates of organ toxicity compared to busulfan or total-body irradiation, albeit with significant mucosal injury. Traditional dosing based on body-surface-area (BSA) may result in non-optimal melphalan exposure among certain patient subsets.

**Objectives:**

As mucosal injury is linked to initiation of alloreactivity, we hypothesized that high exposure of melphalan predicted via a pharmacokinetic (PK) model would be associated with an increased risk of acute graft-versus-host disease (aGVHD).

**Study Design:**

We performed an analysis of 85 patients who underwent A/B-TCD haploidentical HCT on 2 prospective trials using melphalan-based conditioning for treatment of malignancy at 3 centers from 2015 to 2024. Most patients (61.2%) received a total dose of melphalan at 140 mg/m^2^ using actual body weight; others received a dose adjusted for obesity or age <2 years. We analyzed outcomes based on whether melphalan exposure was above or below the median exposure for the group.

**Results:**

The 100-day cumulative incidences of engraftment syndrome (ES), grade II-IV aGVHD, and grade III-IV aGVHD were 34.2%, 24.8%, and 17.1%, respectively. The 3-year cumulative incidence of chronic GVHD (cGVHD), non-relapse mortality (NRM), and relapse were 17.5%, 8.7%, 21.8%, respectively. The 3-year cumulative incidence of disease-free survival (DFS) and severe GVHD-relapse-free survival (GRFS) were 71.4% and 55.6%, respectively. ES was significantly associated with the subsequent development of aGVHD, both grade II-IV (41.4% vs. 17.3% in those with and without ES, *P* = .01) and grade III-IV (34.5% vs. 8.5% in those with and without ES; *P* = .003). Chronic GVHD occurred at significantly higher rates in patients with prior Grade II-IV (66.7% vs. 0% for Grade 0-I; *P* < .001) and Grade III-IV aGVHD (75% vs. 4.3% for Grade 0-II; *P* < .001). Compared to non-obese patients, the PK model predicted lower melphalan exposure (*P* = .02) in obese patients where adjusted ideal body weight was utilized, suggesting overcorrection of the dose. There was no impact of melphalan exposure on immunologic rejection. The median melphalan exposure was 6.81 mg*hr/L (range, 4.4–8.8). Compared to a melphalan exposure ≤6.8 mg*hr/L, a melphalan exposure >6.8 mg*hr/L was associated with a higher incidence of ES (48.8% vs. 19.1%; *P* = .005), grade II-IV aGVHD (39.3% vs. 10.1%; *P* = .002), and grade III-IV aGVHD (31.5% vs. 2.5%; *P* < .001). The 3-year incidence of cGVHD was 27.2% in those with high predicted melphalan exposure compared to 7.4% for low exposure (*P* = .03); with no difference in 3-year NRM incidence (9.2% vs. 7.7%; *P* = .82) or 3-year relapse incidence (16.8% vs. 27.6%; *P* = .31) for high compared to low exposure. However, GRFS was significantly worse in patients with high exposure (46.4%) vs. low exposure (64.6%; *P* = .02).

**Conclusions:**

High melphalan exposure predicted by a validated population PK model is associated with an increased likelihood of developing ES and subsequently acute and chronic GVHD. Given that a substantial number of patients already require adjustment of standard BSA-based dosing for young age or obesity, a prospective trial of model-based dosing to individualize melphalan exposure is warranted to confirm these results.

## INTRODUCTION

Hematopoietic cell transplantation (HCT) is a potentially curative option for certain hematologic malignancies. The availability of well-matched related and unrelated donors is dependent upon the patient’s genetic ancestry [1]. The use of haploidentical related donors broadens donor availability for many patients but is limited by bidirectional alloreactivity that could lead to graft rejection or graft-versus-host disease (GVHD). The recognition that acute GVHD (aGVHD) is primarily mediated by alpha/beta T-cell-receptor (A/B-TCR)-positive T cells led to the development of techniques for their selective elimination from the graft along with CD19+ cells that may serve as a reservoir of Epstein-Barr virus (EBV) and potentially lead to EBV-lymphoproliferative disease [2–6]. Studies of A/B-TCR T-cell depleted (A/B-TCD) HCT performed in Europe demonstrated low rates of GVHD, with grades II-IV acute GVHD (aGVHD) of 14.7%−22%, grades III-IV aGVHD of 0%−6%, and chronic GVHD (cGVHD) of 0%−8.1% [3,5–7].

The US-based PTCTC ONC1401 trial reported a 75% 2-year disease-free survival (DFS) for pediatric patients with acute leukemia or myelodysplastic syndrome (MDS) undergoing A/B-TCD haploidentical HCT [8]. Furthermore, it was noted that patients who received “reduced toxicity” conditioning regimens (with melphalan as the main backbone alkylator) had improved 2-year DFS and overall survival (OS) compared to those who received myeloablative conditioning with busulfan or total body irradiation (TBI) as the backbone agents. An extended dataset of 163 US patients undergoing A/B-TCD haploidentical HCT demonstrated higher rates of severe GVHD than reported in Europe: 25% grade II-IV aGVHD, 8.5% grade III-IV aGVHD, and 19.2% cGVHD [9]. Major differences between the US and Europe that might explain this include: 1) different formulations of rATG (Thymoglobulin^®^ in the US; Grafalon^®^ in the European studies); and 2) different conditioning regimens (melphalan, busulfan, or TBI in the US versus treosulfan, busulfan, or TBI in Europe). Of note, differences in pre- and post-HCT exposure of rabbit anti-thymocyte globulin (rATG) did not impact rates of aGVHD, though lower rates of cGVHD were seen with higher pre-HCT exposure to rATG [9].

Both TBI- and busulfan-based conditioning regimens have been shown to result in increased rates of aGVHD with increasing exposure [10,11]. Melphalan is an alkylating chemotherapy agent with intense myelosuppressive activity that has been widely used in conditioning regimens due to its relatively favorable organ toxicity profile compared to busulfan or TBI [12]. However, melphalan can result in significant, dose-dependent mucosal damage, which is clinically observable as oral mucositis [13–15]. Conditioning-induced gastrointestinal mucosal barrier injury is thought to be the initial step in the cascade leading to the development of GVHD [16,17], potentially via extracellular release of mitochondria and stimulation of antigen-presenting cells [18], amongst other mechanisms [19,20]. Furthermore, it has been demonstrated that rates of aGVHD are higher with prolonged oral mucositis, likely reflecting tissue injury throughout the gastrointestinal tract [13].

Several models exist to describe the pharmacokinetics (PK) of melphalan, based on body weight and creatinine clearance [21–23]. We hypothesized that higher rates of aGVHD seen in US patients undergoing A/B-TCD haploidentical HCT may be partly explained by high exposures of melphalan resulting in subsequent gastrointestinal tissue injury. Accordingly, we performed this analysis with the primary aim of assessing the relationship between clinical outcomes and model-predicted melphalan exposure.

## METHODS

### Study Design and Participants

We performed an unplanned *post hoc* evaluation of patients with hematologic malignancies undergoing HCT using a haploidentical related donor with A/B-TCD on 1 of 2 prospective trials of *ex vivo* T cell depletion: 1) ONC1401 for AB-TCD (NCT02646839; IDE#16412; M. Pulsipher); and 2) PBMT1902 for AB-TCD (NCT04337515; IDE#19096; C. Dvorak), with a data cutoff of 01/01/2025. The initial results of ONC1401 and the combined dataset have been previously published [8,9]. Patients not receiving melphalan as part of conditioning were excluded. The trials were approved by local Institutional Review Boards and informed consent was obtained from patients/parents in accordance with the Declaration of Helsinki.

### Area-Under-the-Curve (AUC) Estimation

Melphalan-based conditioning was administered at a total dose of 140 mg/m^2^ administered over 2 days; patients <2 years of age or <10 kg received 2.33 mg/kg/dose using the “rule of 30.” Patients who were >120% of ideal body weight (IBW) (calculated with the Traub formula [24] for <18 years and the Devine formula [25] for ≥18 years) were dosed by adjusted ideal body weight (AIBW) using the formula: IBW + [0.4 × (actual body weight – IBW)].

Melphalan was combined with fludarabine ± clofarabine, thiotepa (5 mg/kg × 2 doses), and rATG. Some patients received model-based dosing of fludarabine to achieve a goal cAUC exposure of 20 mg*hr/L [26–28], and some received model-based dosing of rATG to achieve an optimized pre- and post-HCT exposure of >50 AU*day/mL and <12 AU*day/mL, respectively [9,29]. Beginning in mid-2020, patients on PBMT1902 received tocilizumab (12 mg/kg for patients <30 kg, 8 mg/kg for those ≥30 kg) on Day −1 to prevent engraftment syndrome (ES), thought to be partly driven by IL-6 [30–33].

Cumulative area-under-the-curve (cAUC, mg*hr/L) estimates for melphalan exposure were obtained using a validated population PK model previously developed by Li et al., [21] and recently updated with additional PK data available from 31 new pediatric HCT recipients (including autologous and allogeneic patients). Complete details of the re-estimation process can be found in the Supplementary Data; and through the InsightRX website (www.InsightRX.com, San Francisco, CA). Briefly, the updated population PK model now incorporates a fat-free mass component and creatinine clearance (capped at 120 ml/min/1.73m^2^) as significant covariates on melphalan clearance. The updated model confirms that the original model was largely unbiased; however, optimization ensures that bias would be even smaller in the exposure-response analysis. Since no concentration data were available for the 85 patients in the exposure-response analysis, population predictions based on patient characteristics included in the updated model were used.

Minimal residual disease (MRD) status was determined by best-available techniques for the patient’s disease at the time, including multiparameter flow cytometry and next-generation sequencing (NGS) of the B and T cell receptors (Adaptive Biotech); [34] patients with lymphomas did not have MRD performed. Body mass index was analyzed according to age-based CDC guidelines in patients ≥2 years of age and classified as underweight, normal, overweight, or obese.

### Outcomes

Patients with graft rejection were censored at the time of rejection for all outcomes other than overall survival. The primary endpoints for analysis were Day 100 cumulative incidence of aGVHD and 3-year cumulative incidence of cGVHD graded and staged by standard criteria [35,36], with relapse and death as competing events. Other secondary outcomes included: 1) Day 100 engraftment syndrome, defined as fevers without identifiable cause starting within 48 hours of appearance of white blood cells and persisting until initiation of hydrocortisone (typically 0.5–1 mg/kg/dose q8hrs) with death as a competing event; 2) 3-year non-relapse mortality (NRM), defined as death from a cause other than relapse, with relapse as a competing event; 3) 3-year relapse, with NRM as a competing event; 4) 3-year relapse-free survival (RFS), defined as the time from HCT to either relapse of hematologic malignancy or death from a cause other than relapse; 5) 3-year GVHD–free relapse-free survival (GRFS), in which events were defined as the first among Grade III–IV aGVHD, severe cGVHD, relapse and death; [37,38] and 6) overall survival (OS). Maximal grade of oral mucositis was not routinely scored or captured.

### Statistical Analysis

Patients were stratified into low vs. high predicted melphalan exposure groups based on the median value for the entire cohort. Furthermore, optimal cutoff exposure associated with Grades II-IV and III-IV aGVHD were identified using receiver operating characteristic (ROC) curves with the maximum Youden’s J statistic. Demographic and disease-related variables were described with the use of frequencies for categorical variables and medians and ranges for quantitative variables. Associations between variables were assessed using Fisher exact test for categorical variables and the Wilcoxon-Mann-Whitney test (for 2 groups) or Kruskal-Wallis (for >2 groups) for continuous variables. Time-to-event endpoints were predicted by using a Kaplan-Meier estimator using log-rank tests for significance. Due to the small sample size, an *a priori* decision was made to only perform bivariate analysis for all outcomes without multivariate analysis upon the recommendation of statistical consultation. Cox regression models using backward selection and likelihood ratios were built using predicted melphalan exposure compared to those covariates with a univariate p-value <.1; for GVHD, NRM, and relapse we used Fine-Grey competing risk models. Statistical analyses were performed with SPSS^®^ Statistics version 29.0 (IBM, Armonk, NY).

## RESULTS

### Patient and Transplant Characteristics

The dataset included 85 patients who underwent haploidentical HCT with melphalan-based conditioning for treatment of a hematologic malignancy from December 2015 to June 2024 at 3 centers (Table 1). Median age at HCT was 10.2 years (range, 0.4–26.5). The group was racially diverse (21.2% White/non-Hispanic), reflecting the disparity in HLA-matched donor availability for minority populations. The median infused cell doses for CD34^+^ and AB-T-cells were 18.7 × 10^6/kg (range, 5–30.8) and 7.5 × 10^4/kg (range, 0.3–10.2), respectively. Median follow-up of survivors was 3.9 years (range, 0.5–9.1).

### Melphalan Predicted Exposures

**Figure 1.**
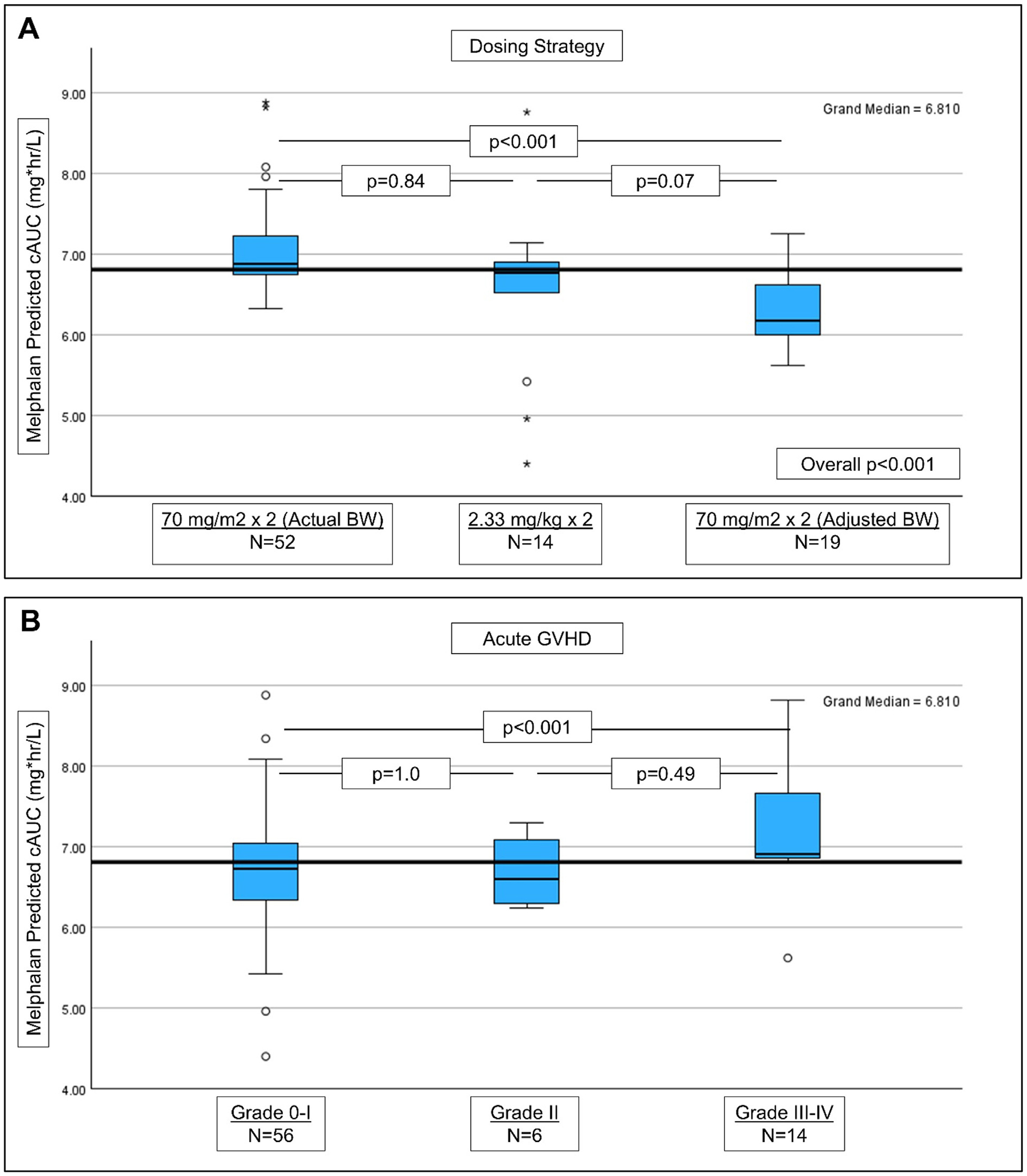
(A) PK-model predicted cAUC by dosing strategy (traditional BSA-based using actual body weight vs. mg/kg-based for infants vs. BSA-based using adjusted body weight for obese patients). (B) PK-model predicted cAUC by development of acute GVHD (Grade 0-I vs. Grade II vs. Grade III-IV).

Most patients (61.2%) received standard 140 mg/m^2^ dosing based on actual body weight; followed by 22.3% of patients dosed by AIBW for >120% of IBW and 16.5% modified for age <2 years. The median predicted melphalan exposure for the entire group was 6.81 mg*hr/L (range, 4.4–8.88), with variable exposure noted by dosing scheme (Figure 1A). Differences in median predicted melphalan exposure (Supplementary Table 1) were seen in patients <10 years of age (6.87 mg*hr/L) compared to those ≥10 years of age (6.54 mg*hr/L; *P* = .03; Supplementary Figure 1a) and by BMI (Supplementary Figure 1B), with obese patients having the lowest predicted exposure (overall *P* = .02), due to overcorrection when using AIBW formulas. In addition, patients receiving haploidentical sibling donor grafts had lower predicted melphalan exposure (Supplementary Figure 1C), likely related to the older age in these recipients (15.8 years vs. 5.9 years for parental donors; *P* = .003).

### Rejection

The Day 100 incidence of graft rejection was 10.6% (95% CI, 4.1%−17.1%). There was no difference in median predicted melphalan exposure in patients who did (6.81 mg*hr/L, range 5.90–7.80) and did not (6.81 mg*hr/L, range 4.40–8.88) experience rejection (*P* = .97; Supplementary Figure 2a) nor any difference in the Day 100 cumulative incidence of rejection between low and high predicted melphalan exposure (*P* = .76; Table 2).

### Engraftment Syndrome

**Figure 2.**
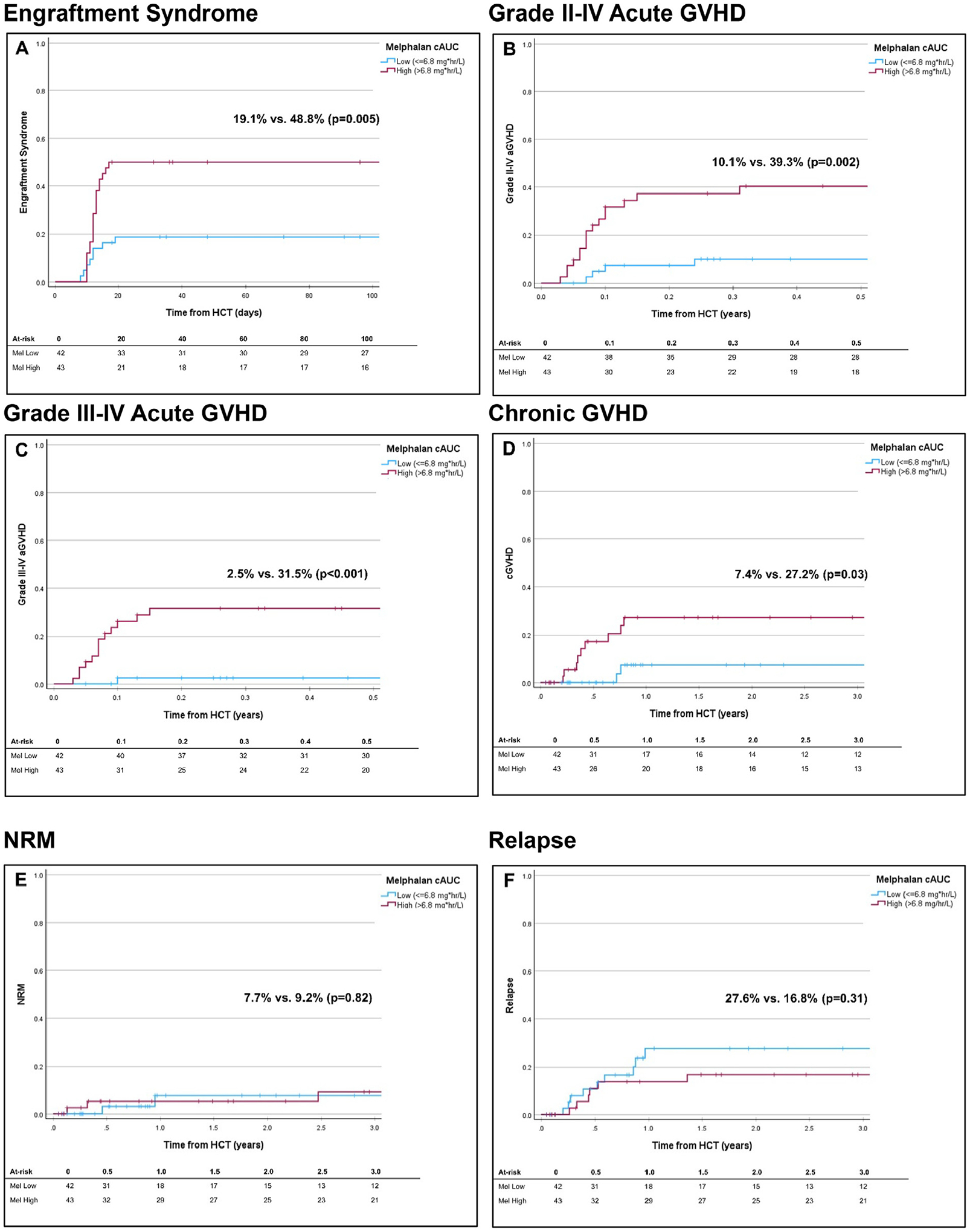
(A) 100-day cumulative incidence of engraftment syndrome by low (≤6.8 mg*hr/L) vs. high (>6.8 mg*hr/L) pre dicted melphalan cAUC. (B) 100-day cumulative incidence of Grade II-IV acute GVHD by low (≤6.8 mg*hr/L) vs. high (>6.8 mg*hr/L) predicted melphalan cAUC. (C) 100-day cumulative incidence of Grade III-IV acute GVHD by low (≤6.8 mg*hr/L) vs. high (>6.8 mg*hr/L) predicted melphalan cAUC. (D) 3-year cumulative incidence of chronic GVHD by low (≤6.8 mg*hr/L) vs. high (>6.8 mg*hr/L) predicted melphalan cAUC. (E) 3-year cumulative incidence of non-relapse mortality by low (≤6.8 mg*hr/L) vs. high (>6.8 mg*hr/L) predicted melphalan cAUC. (F) 3-year cumulative incidence of relapse by low (≤6.8 mg*hr/L) vs. high (>6.8 mg*hr/L) predicted melphalan cAUC.

The 100-day cumulative incidence of ES was 34.2% (95% CI, 24.2–44.2%). On univariate analysis, unknown MRD status (diagnosis of lymphoma), low and high A/B T-cell doses, and lack of tocilizumab were associated with higher incidence of ES (Table 2 and Supplementary Table 2a). Excluding those with graft rejection, there was a higher median predicted melphalan exposure in patients who did (6.92 mg*hr/L; range, 4.4–8.88) compared to those who did not (6.7 mg*hr/L; range, 4.96–8.82) develop ES (*P* = .02; Supplementary Figure 2B). Patients with a predicted melphalan exposure of ≤6.8 mg*hr/L had a Day 100 cumulative incidence of ES of 19.1% (95% CI, 0.7–18.3%) compared to 48.8% (95% CI, 33.9–63.7%) for those with an exposure of >6.8 mg*hr/L (*P* = .005; Figure 2a), resulting in a HR of 2.94 (1.3–6.66; *P* = .01). Bivariate analyses incorporating predicted melphalan exposure retained high melphalan exposure as a risk factor in all pairings, along with unknown MRD status, low and high A/B T-cell doses, and lack of tocilizumab (Supplementary Table 2b).

### Acute Graft-Versus-Host Disease

The 100-day cumulative incidence of Grade II-IV aGVHD was 24.8% (95% CI, 15.4–34.2%). The median onset of aGVHD was Day +30 (range, Day +14–87). In engrafted patients, Grade II-IV aGVHD was significantly more common in patients with preceding ES: 41.4% (95% CI, 24.5%−61.3%) compared to 17.3% (95% CI, 5.5–29.1%) in those without ES (*P* = .01; Supplementary Figure 3a).

On univariate analysis (Supplementary Table 3a), donor type was associated with a differential incidence of Grade II-IV aGVHD: 37.2% (95% CI, 19.6%−54.8%) for maternal, versus 10% (95% CI, 0.1–23.1%) for paternal donors and 19.3% (95% CI, 5.4%−33.2%) for sibling donors (*P* < .001). Excluding those with graft rejection, the median predicted melphalan exposure was higher in patients who did (6.9 mg*hr/L; range, 5.62–8.82) compared to those who did not (6.74 mg*hr/L; range, 4.4–8.88) develop grade II-IV aGVHD (*P* = .047; Supplementary Figure 2c). Patients with a predicted melphalan exposure of ≤6.8 mg*hr/L had a Day 100 cumulative incidence of grade II-IV aGVHD of 10.1% (95% CI, 0.7%−19.5%) compared to 39.3% (95% CI, 24.2–54.4) for those with an exposure of >6.8 mg*hr/L (*P* = .002; Figure 2B), resulting in a HR of 4.76 (1.59–14.25; *P* = .005) for high melphalan exposure. Bivariate analyses incorporating predicted melphalan exposure with the other factors associated with Grade II-IV aGVHD on univariate analysis removed HCT number (first vs. second) and donor type, retaining only high melphalan exposure (Supplementary Table 3b).

The 100-day cumulative incidence of Grade III-IV aGVHD was 17.1% (95% CI, 8.9%−25.3%). Grade III-IV aGVHD was significantly more common in patients with preceding ES: 34.5% (95% CI, 17.3%−51.7%) compared to 8.5% (95% CI, 0.5%−16.5%) in those without ES (*P* = .003; Supplementary Figure 3b). As shown in Supplementary Table 4a, on univariate analysis Grade III-IV aGVHD was associated with maternal donors (*P* < .001), and second HCT (*P* = .009). Excluding those with graft rejection, the median predicted melphalan exposure was higher in patients who did (6.91 mg*hr/L; range, 5.62–8.82) compared to those who did not (6.74 mg*hr/L; range, 4.4–8.88) develop grade III-IV aGVHD (*P* = .001; Supplementary Figure 2d). Furthermore, while the median predicted melphalan exposure for Grade II aGVHD did not differ from Grade 0–1, there was a significant difference when comparing Grade 0-I aGVHD to Grade III-IV (*P* < .001; Figure 1B). Patients with a predicted melphalan exposure of ≤6.8 mg*hr/L had a Day 100 cumulative incidence of grade III-IV aGVHD of 2.5% (95% CI, 0.1%−7.4%) compared to 31.5% (95% CI, 17.2%−45.8%) for those with an exposure of >6.8 mg*hr/L (*P* < .001; Figure 2C), resulting in a HR of 15.1 (1.97–115.54; *P* = .009) for high melphalan exposure. Bivariate analyses incorporating predicted melphalan exposure with the other factors associated with Grade III-IV aGVHD on univariate analysis removed HCT number, donor type, and use of tocilizumab, retaining only high melphalan exposure (Supplementary Table 4b).

### Chronic GVHD

The 3-year cumulative incidence of cGVHD was 17.5% (95% CI, 8.1%−26.9%). The median onset of cGVHD was 0.53 years post-HCT (range, 0.21–0.79). Excluding those with graft rejection, cGVHD was significantly more common in patients with preceding Grade II-IV aGVHD: 66.7% (95% CI, 43.4%−90%) compared to 0% (95% CI, 0%−7.7%) in those with Grade 0-I aGVHD (*P* < .001; Supplementary Figure 3C). Similarly, 3-year cGVHD in those with preceding Grade III-IV aGVHD was 75% (95% CI, 50.5%−99.5%) compared to 4.3% (95% CI, 0.1%−10.2%) in those with Grade 0-II aGVHD (*P* < .001, Supplementary Figure 3d). Grade II aGVHD only was rare and was associated with intermediate risk of developing cGVHD at 50% (95% CI, 1%−99%; Supplementary Figure 3E). The 3-year incidence of cGVHD was higher in patients with maternal donors: 28.9% (95% CI, 9.1%−48.7%) compared to 14.3% (95% CI, 0.1%−32.7%) for paternal donors and 8% (95% CI, 0.1%−18.6%) for haploidentical sibling donors (*P* < .001; Supplementary Table 5a). The use of prophylactic tocilizumab was associated with a lower incidence of cGVHD: 7.9% (95% CI, 0.1%−16.5%) compared to 33% (95% CI, 14.6%−51.4%) for no tocilizumab (*P* = .02).

Patients with a predicted melphalan exposure of ≤6.8 mg*hr/L had a 3-year cumulative incidence of cGVHD of 7.4% (95% CI, 0.1%−17.2%) compared to 27.2% (95% CI, 11.9%−42.5%) for those with an exposure of >6.8 mg*hr/L (*P* = .03; Figure 2D), resulting in a HR of 4.7 (1.02–21.8; *P* = .048) for high melphalan exposure. Bivariate analyses incorporating predicted melphalan exposure with the other factors associated with cGVHD on univariate analysis did not retain any factor as being significant (Supplementary Table 5b). However, the highest risk for cGVHD (43.3%) was seen in those with both high melphalan exposure and lack of tocilizumab, with all other combinations <15% cumulative incidence (*P* = .009; Supplementary Figure 3f).

### NRM and Relapse

The 3-year cumulative incidence of NRM was 8.7% (95% CI, 1.1%−16.3%) at a median of 0.46 years (range, 0.13–2.47) from HCT. As shown in Supplementary Table 6a, there were only 5 NRM events, caused by cGVHD (n=2), acute respiratory failure of unknown etiology (n = 2), and fludarabine neurotoxicity (n = 1). Perhaps due to the low number of events and heterogenous nature of NRM, no baseline patient or HCT characteristic was associated with NRM, including predicted melphalan exposure (*P* = .82; Figure 2E; Supplementary Table 6B). However, in engrafted patients, there was higher 3-year NRM in patients with prior grade II-IV aGVHD: 22.4% (95% CI, 2.8%−42%) compared to 2% (95% CI, 0.1%−5.9%) in those without (*P* = .01; Supplementary Figure 4A).

The 3-year cumulative incidence of relapse was 21.8% (95% CI, 12–31.6%) at a median of 0.45 years (range, 0.2–1.36) from HCT. The only association with relapse was seen in patients with pre-HCT MRD positive disease: 50.8% (95% CI, 25.3%−76.3%) compared to 14.2% (95% CI, 4.4%−24%) for MRD-negative patients, and 0% (95% CI, 0%−48.9%) for MRD unknown patients (*P* = .004; Supplementary Table 7). The hazard ratio (HR) for relapse for patients with MRD-positive disease was 4.32 (1.55–11.99; *P* = .005). There was no significant impact of predicted melphalan exposure on relapse (*P* = .31; Figure 2F), even when controlling for MRD status (Table 2). In engrafted patients, the 3-year relapse rate was lower in those with prior grade II-IV aGVHD: 5.3% (95% CI, 0.1%−15.3%) compared to 27.7% (95% CI, 15.2%−40.2%) in those with grade 0-I aGVHD (*P* = .06; Supplementary Figure 4b). Similarly, 3-year relapse after grade III-IV aGVHD was 0% (95% CI, 0%−25.2%) compared to 26.7% (95% CI, 14.9%−38.5%) in those with grade 0-II aGVHD (*P* = .049; Supplementary Figure 4c). At the time of data cutoff, in the 15 patients with relapse events, only 5 had expired from relapsed disease, with the others either continuing therapy or in remission following CAR-T or subsequent HCT.

### RFS, GRFS, and OS

**Figure 3.**
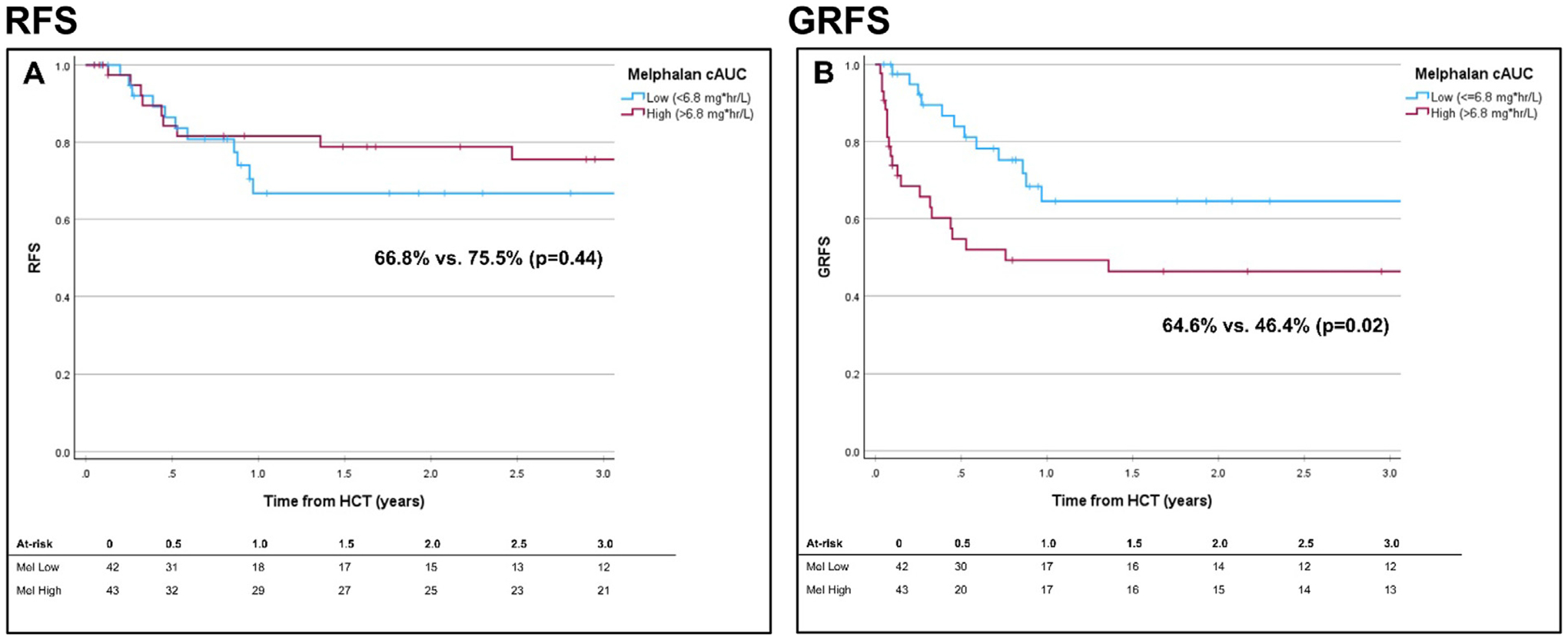
(A) 3-year cumulative incidence of relapse-free survival by low (≤6.8 mg*hr/L) vs. high (>6.8 mg*hr/L) predicted melphalan cAUC. (B) 3-year cumulative incidence of severe GVHD-free relapse-free relapse by low (≤6.8 mg*hr/L) vs. high (>6.8 mg*hr/L) predicted melphalan cAUC.

The 3-year RFS was 71.4% (95% CI, 60.6%−82.2%). On univariate analysis, only pre-HCT MRD-positive status impacted on RFS: 45.4% (95% CI, 20.9%−69.9%) compared to 80.4% (95% CI, 69.4%−91.4%) for MRD-negative and 75% (95% CI, 32.5%−99.9%) for MRD unknown (*P* = .02; Supplementary Table 8). The HR for relapse for patients with MRD-positive disease was 3.41 (1.38–8.44; *P* = .008). There was no significant impact of predicted melphalan exposure on RFS (*P* = .44; Figure 3A), even when controlling for MRD status (Table 2).

The 3-year GRFS was 55.6% (95% CI, 44–67.2%). On univariate analysis, the most important factor for GRFS was donor type, with 71.7% (95% CI, 54.8–88.6%) for sibling donors compared to 52.7% (95% CI, 26–79.4%) for paternal donors and 40% (95% CI, 21.6–58.4%) for maternal donors (*P* < .001; Supplementary Table 9a). Patients with predicted melphalan exposure >6.8 mg*hr/L had a 3-year GRFS of 46.4% (95% CI, 30.5–62.3%) compared to 64.6% (95% CI, 48.1–89.6%) for melphalan cAUC ≤6.8 mg*hr/L (*P* = .02). This association was most noticeable for patients with pre-HCT MRD-negative status, where the 3-year GRFS for predicted melphalan exposure >6.8 mg*hr/L was 44.4% (95% CI, 26–62.8%) compared to 71% (95% CI, 52.4–89.6%) for melphalan cAUC ≤6.8 mg*hr/L (*P* = .02; Table 2). However, bivariate analysis revealed that not using a sibling haploidentical donor was a risk factor for worse GRFS (Supplementary Table 9b).

The 3-year OS was 84.1% (95% CI, 75.7–92.5%). Because many patients with relapse were salvaged with additional therapy beyond the scope of this report, no additional analysis was performed on factors associated with OS. There was no difference in OS by predicted melphalan exposure: >6.8 mg*hr/L had a 3-year OS of 80% (95% CI, 67.5–92.5) compared to 89.7% (95% CI, 80.1–99.3) for melphalan cAUC ≤6.8 mg*hr/L (*P* = .36).

### Melphalan Optimal Exposure Range Estimation

ROC curve analyses showed that the best predicted melphalan exposure values to predict development of Grades II-IV and III-IV aGVHD were both 6.81 mg*hr/L (80% sensitivity, 60.7% specificity for Grades II-IV and 92.9% specificity and 57.3% sensitivity for Grades III-IV; Supplementary Figures 5a and 5b), validating the *a priori* decision to use the median predicted exposure for the dichotomous analyses. However, as previously noted, when using the median predicted cAUC as a cut-off, RFS was non-significantly lower and GRFS was significantly higher with low melphalan exposure (Figure 3). We therefore further hypothesized that the optimal outcomes would be associated with predicted melphalan exposures just below the threshold that is associated with significant alloreactivity and severe aGVHD, but potentially high enough to cause sufficient tissue injury to mediate the initiation of a graft-versus-leukemia effect.

While the dichotomous analyses using the medians was a robust discriminator, we therefore performed an exploratory analysis by dividing the patients into 3 groups of melphalan based on visual assessment of Figure 1B and examining the exposures that maintained the highest Youden’s Indices on the ROC curves for aGVHD: Low (cAUC <6.7 mg*hr/L), Medium (cAUC 6.7–6.84 mg*hr/L); and High (cAUC ≥6.85 mg*hr/L). As summarized in Supplementary Table 10, although the Medium predicted exposure group was small (n = 13) and therefore underpowered, this group had low rates of ES and Grade II-IV aGVHD (Supplementary Figures 6a and 6b). The incidences of Grade III-IV aGVHD and cGVHD in the Medium predicted exposure group were intermediate between the Low and High groups (Supplementary Figures 6c and 6d). RFS were similar in the Medium and High Groups (Supplementary Figure 6E). GRFS was apparently optimized for the Medium Group (Supplementary Figure 6f), suggesting that this may be a goal exposure window to target in a confirmatory trial.

## DISCUSSION

This analysis demonstrates that predicted exposure of melphalan is associated on univariate analysis with the development of engraftment syndrome and GVHD following A/B-TCD haploidentical HCT in patients with malignant disease. Melphalan is used to eliminate host bone marrow hematopoietic stem cells during HCT conditioning, and as expected our analysis showed that predicted melphalan exposure played no role in the risk of graft rejection, a process which is instead more likely most impacted by exposure of immunoablative agents, such as rATG, fludarabine and thiotepa [26,27,39]. We did note on univariate analysis a strong association between high predicted melphalan exposure and subsequent development of engraftment syndrome, which we hypothesize is due to increased mucosal injury. We did not rigorously collect clinical grading of oral mucositis; however, this may be a poor surrogate for intestinal mucositis, which is more challenging to grade. Novel biomarkers, such as blood citrulline levels, may someday be useful in this regard [40]. The pathophysiology of ES is not well understood, but it has been proposed to be mediated by activated macrophages producing proinflammatory cytokines such as IL-6, IL-12, and IL-1*β* [33].

Given the hyper-cytokine state that occurs with ES, it is not surprising that aGVHD was significantly more common in patients with preceding ES. The subsequent association of high predicted melphalan exposure with grade II-IV and grade III-IV aGVHD, as well as cGVHD, suggests that potential of targeted melphalan exposure through model-based dosing to limit excessive exposure may help prevent severe GVHD. Surprisingly, there was no relationship between predicted melphalan exposure and NRM. This analysis was limited by a small number of NRM events; however, it may also be impacted by the fact that only 2 patients died secondary to GVHD. Nevertheless, this analysis suggests that melphalan-based conditioning is a generally well-tolerated approach and that optimization of other factors in the HCT approach is likely more important to the achievement of further reduction in NRM. Prevention of severe aGVHD is a worthwhile goal given the increased risk of developing cGVHD and it’s significant impact on quality-of-life, as well as the association of GVHD with NRM [41,42]. However, in patients with malignant disease, this must be done with caution to not abrogate the GVL effect and increase relapse, especially in the highest-risk (MRD-positive) patients. While relapse incidences were lower in patients with Grade II-IV and Grade III-IV aGVHD, RFS was not statistically superior in the higher predicted melphalan exposure group. It should also be noted that when using GRFS as the endpoint, patients with lower predicted melphalan exposure had statistically better outcomes, especially for patients with pre-HCT MRD-negative status. Although this analysis was performed solely in patients undergoing A/B-TCD haploidentical HCT for the treatment of a malignant condition, there is no biologic reason to suspect that this finding would not also apply to patients with non-malignant conditions, where there is little benefit of developing GVHD. Of note, a recent analysis of adult patients with AML/MDS using melphalan-based conditioning similarly demonstrated significantly higher rates of Grade II-IV aGVHD and cGVHD with higher doses of melphalan [43].

Over a third of patients in this cohort did not receive standard BSA-based dosing due to age <2 years or obesity. Melphalan is transported into the cells by amino acid transport systems and within the cell melphalan undergoes chemical hydrolysis; the metabolites are excreted through the kidney into the urine with a biological half-life of approximately sixty minutes. As such, patients with poor renal function likely benefit from adjusted dosing. We suggest that PK-driven, model-based dosing (MBD) incorporating fat-free mass and renal function as covariates is a superior method to BSA-based dosing for achieving consistent and optimal exposure of melphalan over a continuous range of weights often seen in pediatric studies, where there is no standard approach for how to adjust for obesity [44], and is subject to “cliff-effects” whereby a slight increase in age or weight may tip someone from the “not-adjusted” to “adjusted” category (or vice versa) and produce significant variations in prescribed dose (examples provided in Supplementary Table 11).

Melphalan-based conditioning has previously been associated with excellent RFS in the setting of A/B-TCD haploidentical HCT for malignant disease [8], and a larger dataset of 163 patients with hematologic malignancies showed no significant differences in 3-year NRM, relapse, or RFS between melphalan-, busulfan-, or TBI-based regimens [9]. However, severe GVHD is common, and the present analysis suggests that optimization of melphalan exposure may lead to improved HCT outcomes. Exploratory analyses suggest that use of MBD to target a predicted melphalan exposure just below the median of the whole population (6.8 mg*hr/L) may be associated with acceptable incidences of ES, aGVHD, and cGVHD, while optimizing RFS and GRFS. We plan to prospectively validate the findings of this study in a follow-up trial of A/B-TCD haploidentical HCT for both malignant and non-malignant conditions.

This study has several limitations. First, the melphalan exposures studied in this cohort were predicted from a PK model based on and patient characteristics alone (i.e. population predictions), and not based on measured concentrations. As such, there is unaccounted variability in the actual exposure experienced by an individual patient, and some patients on the border of the cutoff level of a cAUC of 6.8 mg*hr/L may have been incorrectly characterized as either high or low, although we would not expect structural bias given that the model was optimized for this population. Second, we did not have a direct or surrogate biomarker for measurement of mucosal damage, so the putative mechanism for these findings remains speculative. Third, other unmeasured factors, such as perturbations in the gastrointestinal microbiome [45], could also be involved in the pathogenesis of GVHD. Fourth, while this represents 1 of the largest cohorts of patients receiving melphalan-based conditioning for an A/B-TCD haploidentical HCT, the absolute number of patients may have limited the power to detect some differences, especially in relapse incidence between different types of malignant diagnoses. The small cohort also precluded a multivariate analysis that might have accounted for underappreciated confounding factors such as donor source or prior HCT. Finally, any findings regarding the association of high predicted melphalan exposure with ES and aGVHD in the setting of A/B-TCD haploidentical HCT may not translate to T-replete HCT approaches; these would need to be studied separately.

In conclusion, we demonstrate that, in the A/B-TCD haploidentical HCT setting, standard BSA-based dosing of melphalan leads to variable predicted exposures. In turn, high predicted melphalan exposure is linked to the development of ES, aGVHD, and cGVHD, as well as worse GRFS, especially in MRD-negative patients. We hypothesize that high melphalan exposure leads to increased mucosal injury, which in turn results in increased expression by activated macrophages of pro-inflammatory cytokines such as TNF-a, IL-6, and IL-1b. These pyrogenic cytokines then bind to receptors in the hypothalamus triggering the release of prostaglandins that increase body temperature, manifesting as ES, and initiate the development of alloreactivity in the form of acute GVHD. We plan to prospectively validate these findings in a study where MBD of melphalan (targeting an exposure of 6.8 mg*hr/L) is utilized along with MBD of other agents. Precisely individualizing the exposure of the various components of the conditioning regimen not only holds the potential for improved outcomes, but also standardizes this part of the HCT process, thereby facilitating improved analysis of other factors (e.g., donor characteristics, cell doses, etc.) that might impact on major outcomes.

## Supplementary Material

1

2

3

Supplementary material associated with this article can be found in the online version at doi:10.1016/j.jtct.2025.03.020.

## Figures and Tables

**Table 1:** Patient Demographics and Transplant Characteristics by Predicted Melphalan Exposure

Patient or Transplant Factor	Overall	Melphalan Low Exposure (≤6.8 mg*hr/L)	Melphalan High Exposure (>6.8 mg*hr/L)	P value
N	85	42	43	-
Age				<.001
<10 years	42 (49.4%)	13 (31%)	29 (67.4%)	
≥10 years	43 (50.6%)	29 (69%)	14 (32.6%)	
Sex				.12
Male	51 (60%)	29 (69%)	22 (51.2%)	
Female	34 (40%)	13 (31%)	21 (48.8%)	
Race & Ethnicity				.42
White/Non-Hispanic	18 (21.2%)	8 (19%)	10 (23.3%)	
White/Hispanic	41 (48.2%)	23 (54.8%)	18 (41.9%)	
Black	8 (9.4%)	2 (4.8%)	6 (14%)	
Asian	18 (21.2%)	9 (21.4%)	9 (20.9%)	
Body Mass Index (per CDC)				.01
Not done (<2 years old) [1]	14 (16.5%)	8 (19%)	6 (14%)	
Underweight	6 (7.1%)	2 (4.8%)	4 (9.3%)	
Healthy Weight	43 (50.6%)	15 (35.7%)	28 (65.1%)	
Overweight	8 (9.4%)	5 (11.9%)	3 (7%)	
Obese [2]	14 (16.5%)	12 (28.6%)	2 (4.7%)	
Creatinine Clearance				.11
<120 ml/min/1.73m^2^	16 (18.8%)	5 (11.9%)	11 (25.6%)	
≥120 ml/min/1.73m^2^	69 (81.2%)	37 (88.1%)	32 (74.4%)	
Disease				.85
ALL	48 (56.5%)	25 (59.5%)	23 (53.5%)	
AML/MDS	24 (28.2%)	11 (26.2%)	13 (30.2%)	
Other	13 (15.3%)	6 (14.3%)	7 (16.3%)	
MRD Status				.8
Negative (by NGS or Flow)	60 (70.6%)	29 (69%)	31 (72.1%)	
Positive (by NGS or Flow)	20 (23.5%)	11 (26.2%)	9 (20.9%)	
Not done	5 (5.9%)	2 (4.8%)	3 (7%)	
HCT Number				.03
First	76 (89.4%)	41 (97.6%)	35 (81.4%)	
Second	9 (10.6%)	1 (2.4%)	8 (18.6%)	
Donor [4]				<.001
Mother	32 (37.6%)	10 (23.8%)	22 (51.2%)	
Father	20 (23.5%)	7 (16.7%)	13 (30.2%)	
Sibling (full- or half-)	32 (37.6%)	25 (59.5%)	7 (16.3%)	
Other (child)	1 (1.2%)	0	1 (2.3%)	
Donor Age				.009
<30 years	40 (47.1%)	26 (61.9%)	14 (32.6%)	
≥30 years	45 (52.9%)	16 (38.1%)	45 (52.9%)	
CMV Serostatus				.43
R−/D−	22 (25.9%)	13 (31%)	9 (20.9%)	
R−/D+	17 (20%)	10 (23.8%)	7 (16.3%)	
R+/D+	42 (49.4%)	17 (40.5%)	25 (58.1%)	
R+/D−	4 (4.7%)	2 (4.8%)	2 (4.7%)	
rATG Exposure Optimized [5]				.83
No	51 (60%)	26 (61.9%)	25 (58.1%)	
Yes	34 (40%)	16 (38.1%)	18 (41.9%)	
CD34 Cell Dose, median				.59
<18.7 × 10^6/kg	42 (49.4%)	22 (52.4%)	20 (46.5%)	
≥18.7 × 10^6/kg	43 (50.6%)	20 (47.6%)	23 (53.5%)	
A/B T-Cell Dose, median				.7
<7.5 × 10^4/kg	23 (27.1%)	10 (23.8%)	13 (30.2%)	
7.5 × 10^4/kg	29 (34.1%)	16 (38.1%)	13 (30.2%)	
>7.5 × 10^4/kg	33 (38.8%)	16 (38.1%)	17 (39.5%)	
Tocilizumab Prophylaxis				.13
No	38 (44.7%)	15 (35.7%)	23 (53.5%)	
Yes	47 (55.3%)	27 (64.3%)	20 (46.5%)	

Use of the BMI-for-age growth chart is not recommended for children younger than age 2 years.

Overweight and obese patients were often dosed via adjusted ideal body weight (see Methods for details).

Other diagnoses include: mixed phenotype acute leukemia (n = 8); peripheral T-cell lymphoma (n = 2), large B-cell lymphoma (n = 1), chronic myelogenous leukemia (n=1), and juvenile myelomonocytic leukemia (n = 1).

Patients with parental donors were younger (median age=5.9 years) compared to those with sibling donors (median age=15.8 years; *P* = .003).

Pre-HCT AUC >50 AU*day/mL; Post-HCT AUC <12 AU*day/mL.

**Table 2:** Summary of Univariate Associations of Melphalan Exposure with Clinical Outcomes

		Melphalan Low Exposure (≤6.8 mg*hr/L)		Melphalan High Exposure (>6.8 mg*hr/L)		P value
Outcome	Overall	N events	Cumulative Incidence (95% CI)	N events	Cumulative Incidence (95% CI)	
Graft Rejection (Day 100)	10.6% (4.1–17.1%)	4/42	9.5% (0.7–18.3%)	5/43	11.6% (2–21.2%)	.76
Engraftment Syndrome (Day 100)	34.2% (24.2–44.2%)	8/42	19.1% (7.1–31.1%)	21/43	48.8% (33.9–63.7%)	.005 (1)
No Tocilizumab	50.3% (36.9–69.1%)	5/15	34% (9.7–58.3%)	14/23	60.8% (40.8–80.8%)	.14
Tocilizumab	21.3% (9.5–33.1%)	3/27	11.1% (0.1–22.9%)	7/20	35% (14–56%)	.054
Grade II-IV aGVHD (Day 100)	24.8% (15.4–34.2%)	4/42	10.1% (0.7–19.5%)	16/43	39.3% (24.2–54.4%)	.002 (2)
Grade III-IV aGVHD (Day 100)	17.1% (8.9–25.3%)	1/42	2.5% (0.1–7.4%)	13/43	31.5% (17.2–45.8%)	<.001 (3)
cGVHD (3-year)	17.5% (8.1–26.9%)	2/42	7.4% (0.1–17.2%)	9/43	27.2% (11.9–42.5%)	.03
NRM (3-year)	8.7% (1.1–16.3%)	2/42	7.7% (0.1–18.3%)	3/43	9.2% (0.1–19.4%)	.82
Relapse (3-year)	21.8%(12–31.6%)	9/42	27.6% (11.9–43.3%)	6/43	16.8% (4.5–29.1%)	.31
MRD Negative	14.2% (4.4–24%)	5/29	20.6% (4.1–37.1%)	2/31	8% (0.1–18.8%)	.16
MRD Positive	50.8% (25.3–76.3%)	4/11	50.6% (13.9–87.3%)	4/9	57.1% (20.4–93.8%)	.38
RFS (3-year)	71.4% (60.6–82.2%)	11/42	66.8% (50.3–83.3%)	9/43	75.5% (61.6–89.4%)	.44
MRD Negative	80.4% (69.4–91.4%)	6/29	74.1% (55.9–92.3%)	4/31	82.6% (68.7–96.5%)	.34
MRD Positive	45.4% (20.9–69.9%)	5/11	45% (10.9–79.1%)	4/9	50% (10–90%)	.9
GRFS (3-year)	55.6% (44–67.2%)	12/42	64.6% (48.1–81.1%)	21/43	46.4% (30.5–62.3%)	.02
MRD Negative	57.2% (43.7–70.7%)	7/29	71% (52.4–89.6%)	16/31	44.4% (26–62.8%)	.02
MRD Positive	45.4% (20.9–69.9%)	5/11	45% (10.9–79.1%)	4/9	42.9% (6.2–79.6%)	.54
OS (3-year)	84.1% (75.7–92.5%)	4/42	89.7% (80.1–99.3%)	8/43	80% (67.5–92.5%)	.36
MRD Negative	86.8% (77.4–96.2%)	3/29	88.3% (75.8–99.9%)	4/31	85.8% (72.7–98.9%)	.9
MRD Positive	78.3% (59.3–97.3%)	1/11	90.9% (73.8–99.9%)	3/9	66.7% (35.9–97.5%)	.28

aGVHD, acute graft-versus-host disease; cGVHD, chronic graft-versus-host disease; NRM, non-relapse mortality; MRD, minimal residual disease; DFS, disease-free-survival; GRFS, GVHD-free, relapse-free survival.

Also significant on all bivariate analyses incorporating other factors associated with Engraftment Syndrome (see Supplementary Table 2B).

Also significant on all bivariate analyses incorporating other factors associated with Grade II-IV aGVHD (see Supplementary Table 3B).

[3 Also significant on all bivariate analyses incorporating other factors associated with Grade III-IV aGVHD (see Supplementary Table 4B).
