# Supplementary material for: High Melphalan Exposure Increases the Risk of Graft-Versus-Host Disease in Pediatric Patients Undergoing Alpha-Beta T-Cell Depleted Haploidentical Transplantation
Source: Transplant Cell Ther. Author manuscript; Available in PMC 2026 May 18. (PMC13181854; doi:10.1016/j.jtct.2025.03.020)
Supplement: 1 [file NIHMS2173896-supplement-1.docx]

# Supplementary Data: Model re-evaluation with addition melphalan time-concentration data

# *Background*

# The initial model published by our group included 37 subjects with a median age of 7.3 years (range 1.4 – 17.7).^1^ The goal of this analysis was to re-evaluate the model with *additional* time-concentration data available in 31 pediatric patients obtained through our ongoing PK study of melphalan as part of pre-transplant conditioning prior to hematopoietic cell transplantation (HCT). The re-evaluation process was used to investigate earlier modeling decisions, particularly around covariate relationships given new PK data was available in a more heterogenous group of subjects. The new data set allowed us to better evaluate covariates of interest including maturation, fat-free mass, and renal function on melphalan exposure. Patient demographics of the original and new data sets are provided below in Supplementary Table 1.

The new data set consisted of 31 new subjects for a total of 61 evaluable concentrations. Indication for allogeneic or autologous transplantation was for a variety of malignant and nonmalignant pediatric disorders. Drug levels for melphalan were analyzed by a validated assay as previously described.^2^ A total of 9 (%) samples fell below the limit of quantification and were entered into the analysis as 50 ng/mL (half of the lower limit of quantification of 100 ng/mL).

**Supplementary Table 1:** Patient Demographics of the original, new, and combined dataset. Data are presented as median (range).

| **Patient characteristic** | **Original Dataset**  **(Li et al.)^1^** | **New Dataset (unpublished)** | **Combined Final Dataset** |
| --- | --- | --- | --- |
| Number of patients | 31 | 37 | 68 |
| Sex (male / female) | 15 / 22 | 13 / 18 | 28 / 40 |
| Age (years) | 8.12  (1.47 – 17.7) | 8.3  (0.7 – 22.0) | 8.12  (0.7 – 22.0) |
| Weight (kg) | 22.0  (8.1 – 103.0) | 28.8  (7.66 – 116) | 24.4  (7.66 – 116) |
| Height (cm) | 121  (74.5 – 175) | 134  (68 – 190) | 123  (68 – 190) |
| Serum creatinine (mg/dL) | 0.31  (0.13 – 0.77) | 0.36  (0.15 – 0.95) | 0.35  (0.13 – 0.95) |
| CrCL(ml/min/1.73m2) | 155  (78.2 – 345) | 143  (59.1 – 250) | 147  (59.1 – 345) |

***Original Model Code***

The model by Li et al. was a 2-compartment PK model, with adjusted body weight (AdjBW) as only covariate, used in an allometric context. It was defined as follows (rewritten to NONMEM from original implementation in Pumas):

$PROBLEM
$SUBROUTINES ADVAN6 TOL = 6

$MODEL
NCOMP = 3
COMP = (CENTR) ; central
COMP = (PERIP) ; peripheral
COMP = (AUC) ; AUC

$PK
IBW = HT * HT * 1.65 / 1000
HT_in = HT / 2.54
IF(HT_in.GT.60.AND.SEX.EQ.1) IBW_base = 39
IF(HT_in.GT.60.AND.SEX.EQ.0) IBW_base = 42.2
IF(HT_in.GT.60) IBW = IBW_base + 2.27 * (HT_in - 60)

AJBW = WT
THRESHOLD = 1.2 * IBW
IF(AJBW.GT.THRESHOLD) AJBW = IBW + 0.4 * (WT - IBW)

CL = THETA(1) * (AJBW/25) ** 0.75 * EXP(ETA(1))
V = THETA(2) * (AJBW/25) * EXP(ETA(2))
Q = THETA(3) * (AJBW/25) ** 0.75
V2 = THETA(4) * (AJBW/25) * EXP(ETA(3))

K12 = Q/V
K21 = Q/V2

$DES
DADT(1) = -K12 * A(1) + K21 * A(2) - CL/V * A(1)
DADT(2) = K12 * A(1) - K21 * A(2)
DADT(3) = A(1)/V

$ERROR
IPRED = A(1)/V
PROP = 0.125662
ADD = 0.01
Y = IPRED * (1 + PROP * EPS(1)) + ADD * EPS(2)

$THETA
19.1 ; 1 CL L/h/25kg
8.5 ; 2 V L/25kg
12.4 ; 3 Q L/h/25kg
5.8 ; 4 V2 L/25kg

$OMEGA
0.042712 ; 1 CL
0.047233 ; 2 V
0.07978 ; 3 V2

$SIGMA
1
1

# *Model Re-Evaluation*

# Model evaluation was performed using NONMEM v7.4.1 and the FOCEI estimation method. The covariance step was used to obtain estimates of uncertainty for the parameter estimates. The model re-evaluation occurred in three steps.

## Step 1: Model re-fit based on previous dataset

In this step the original model was refitted on the original dataset. This is a relevant step since a different software was used originally (Pumas), and hence slight differences may be expected.

The fit resulted in very similar parameter estimates as reported in the original article (table 1). The only relevant changes were noticed in the magnitude of inter-individual variability (IIV), which went up by about 12% for CL and 40% for V, while IIV for V2 stayed approximately the same. The fixed effects also differed somewhat. Residual error magnitude was slightly lower in the refit.

## Step 2: Model re-fit based on previous dataset and new data

In this step the original model was refitted on a merged dataset containing the original (n=37 patients) and new data (n=31 patients). No modifications were made to the model in this step.

This resulted in very similar parameter estimates as obtained in step 1, i.e. the new data did not markedly alter the PK estimates. The most marked change were increases in magnitude of IIV for CL (to 42%) and V2 (to 40.1%).

**Supplementary Table 2.** Parameter estimates for the re-evaluated model.

| **Parameter** | **Li et al.  (Pumas)** | **Li Model / Original Data (NONMEM, step 1)** | | | **Li Model / New Data (NONMEM, step 2)** | | |
| --- | --- | --- | --- | --- | --- | --- | --- |
| Clearance (L/h) | 19.1 | 21.1 | 5.7% |  | 17.4 | 5.3% |  |
| Volume of distribution in the central compartment (L) | 8.5 | 9.7 | 14% |  | 7.45 | 15% |  |
| Intercompartmental clearance (L/h) | 12.4 | 14.0 | 17% |  | 13.6 | 15% |  |
| Volume of distribution in the peripheral compartment (L) | 5.8 | 6.6 | 9.1% |  | 6.5 | 6.4% |  |
| Proportional error | 12.6% | 12.4% | 14% | 26% | 12.3% | 14% | 36% |
| Between subject variability on clearance | 20.7% | 32.6% | 17% | 4.3% | 42.5% | 12% | 1.9% |
| Between subject variability on the volume of distribution (central compartment) | 21.7% | 66.3% | 20% | 13% | 67.1% | 16% | 11% |
| Between subject variability on the volume of distribution (peripheral compartment) | 28.2% | 28.7% | 23% | 17% | 40.1% | 21% | 20% |

## Step 3: Structural model re-evaluation

The two covariate effects included in the model were re-evaluated: renal function and body weight.

###

### *Re-Evaluation of Covariates*

### Impact of renal function: Renal function, which was not included in the previous model, was estimated using the Bedside Schwartz equation and was now a significant predictor of CL (p < 0.01, dOFV = -17.6 points). Overall, the effect magnitude was modest. The estimated exponent in the power-relationship was 0.33 (0 = no eﬀect, 1 = linear). This covariate explained ~2.5% of variability in CL (42.5% —> 40%).

- **Body Weight****:** In the previous model, weight was incorporated as adjBW as a covariate on CL. In our evaluation we observed that FFM was a slightly better predictor of CL than AdjBW. Although the improvement was small, this is observed often for other drugs as well (FFM being the best weight-based descriptor), so we altered the model to incorporate FFM as a covariate on CL. This reduced IIV on CL by 0.3% and residual error by 0.1%.

### Age and Maturation: Effects on maturation (AGE) were tested on the PK parameters, but did not improve the fit: no significance was obtained and the effect magnitude was very close to zero.

- No other covariates were available for testing

***Goodness of Fit Plots***

The figures below show the basic goodness of fit plots of population predictions (PRED) and individual predictions (IPRED) versus observed concentrations (DV) for the final model. Also shown are plots of conditional weighted residuals (CWRES) versus population predictions and versus time. All plots indicate that the model has no discernable bias in its predictions.

**Supplementary Figure 1**. Original Model and New Model

***
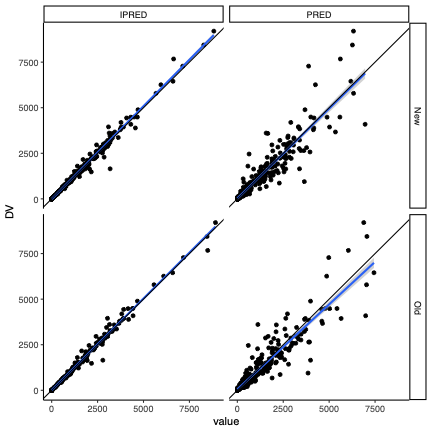
***

*
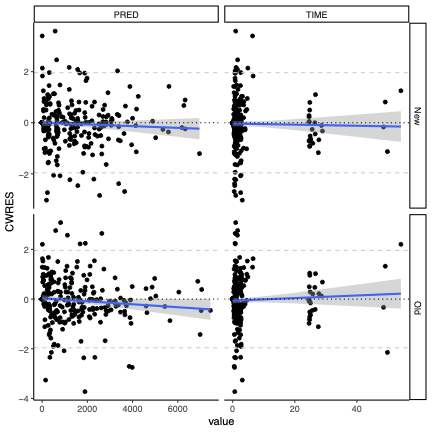
*

**Supplementary Figure 2.** Group by Age

*
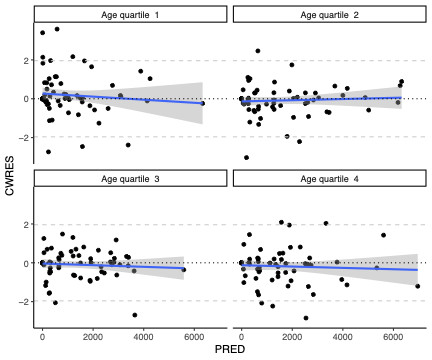
*

*
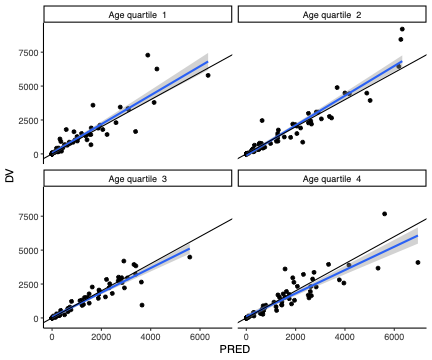
*

**Supplementary Figure 3.** Plots by Gender

*
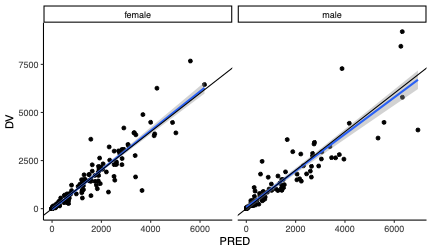
*

***Visual Predictive Check***

The visual predictive checks in the figures below, shown both on the regular scale and on log-scale, show the 5^th^, 50^th^ and 95^th^ percentiles of the observed data (lines) as well as their respective confidence intervals obtained using repeated simulations from the model. This plot also indicates that the model describes both the general trend and the variability in the data well.

**Supplementary Figure 4.** Visual Predictive Check

******

******

***Summary of the Re-Evaluation***

- The original model was unbiased in its fixed parameters.
- CrCL is a moderate/low predictor of melphalan clearance.
- IIV was underestimated in the original model. This is potentially important as this aﬀects MAP estimates (CL, V, AUC), especially with limited sampling, so important for AUC estimation and when used in dose adaptation.
- The final model is unbiased and described the observed data well

# *Final Parameter Estimates of the Re-Evaluation*

**Supplementary Table 3.** Final parameter estimates using FOCEI in NONMEM.

| **Parameter** | **Value** | **Unit** | **RSE** | **Shrinkage** |
| --- | --- | --- | --- | --- |
| Clearance (L/h) | 20.2 | L/hr | 5.9% |  |
| Volume of distribution in the central compartment | 12.1 | L | 15% |  |
| Intercompartmental clearance | 11.6 | L/hr | 30% |  |
| Volume of distribution in the peripheral compartment | 8.24 | L | 6.5% |  |
| Between subject variability on clearance | 54.2 | % | 13% | 3.0% |
| Between subject variability of volume of distribution (central) | 67.1 | % | 19% | 9.2% |
| Between subject variability of volume of distribution (peripheral) | 57.0 | % | 25% | 13.6% |
| Proportional error | 12.7 | % | 15% | 33% |

Notes:

- Covariate effect sizes available in NONMEM code
- IIV % calculated as $e^{\sqrt{\omega^{2}-1}}$

# *NONMEM Final Model Code*

The model code below includes the final parameter estimates. Units for covariates are: serum creatinine (mg/dL), weight (kg), height (cm).

$PROBLEM
$INPUT
$DATA

$SUBROUTINE ADVAN6 TOL=6
$MODEL NCOMP=3 COMP=(CENTR) ; central
 COMP=(PERIP) ; peripheral
 COMP=(AUC) ; AUC
$PK
CRCL = 0.413 * HT/CR
IF(CRCL.LT.120) THEN
 CRCLM = CRCL
ELSE
 CRCLM = 120
ENDIF

BMI = WT/((HT*HT)/10000);
IF(SEX.EQ.0) THEN ; female:
 FFM = (1.11 + ((1-1.11)/(1+((AGE/7.1)**(-1.1))))) * ((9270 * WT)/(8780 + (244 * BMI)))
ELSE ; male:
 FFM = (0.88 + ((1-0.88)/(1+((AGE/13.4)**(-12.7))))) * ((9270 * WT)/(6680 + (216 * BMI)))
ENDIF

TVCL = THETA(1) * (FFM/25) ** 0.75 * (CRCLM/100) ** THETA(7)
CL = EXP(LOG(TVCL) + ETA(1))
TVV = THETA(2) * (FFM/25)
V = EXP(LOG(TVV) + ETA(2))

TVVQ = THETA(3) * (FFM/25) ** 0.75
Q = EXP(LOG(TVVQ))
TVV2 = THETA(4) * (FFM/25)
V2 = EXP(LOG(TVV2) + ETA(3))

K12 = Q/V
K21 = Q/V2

$DES
DADT(1) = -K12 * A(1) + K21 * A(2) - CL/V * A(1)
DADT(2) = K12 * A(1) - K21 * A(2)
DADT(3) = A(1)/V

$ERROR
IPRED = A(1)/V
PROP = THETA(5)
ADD = THETA(6)
Y = IPRED * (1 + PROP * EPS(1)) + ADD * EPS(1)
AUC = A(3)

$THETA
 (0,20.2145) ; CL_Lperhr
 (0,12.1147) ; V_L
 (0,11.5762) ; Q_Lperhr
 (0,8.24349) ; V2_L
 (0,0.127193) ; prop error
 (0,0.001) FIX ; add error
 (0,0.53776) ; CL_CRCL

$OMEGA BLOCK(3)
 0.150099 ; IIV_CL
 0.200752 0.360378 ; IIV_V
 0.112007 0.213578 0.192386 ; IIV_V2

$SIGMA 1 FIX

**References:**

1. Li, Shuhui, Dvorak, C, Lu, Ying, et. al., Population pharmacokinetics of melphalan in pediatric patients undergoing hematopoietic cell transplantation. Journal of Clinical Pharmacology. 2022 Jul; 62(7): 873-882.

2. Huang, L, Cheah, V, Chan, D, et. al., Determination of melphalan in human plasma by UPLC-UV method. Cancer Chemotherapy and Pharmacology. 2019 Feb; 83: 905-910.
