# Supplementary material for: High Melphalan Exposure Increases the Risk of Graft-Versus-Host Disease in Pediatric Patients Undergoing Alpha-Beta T-Cell Depleted Haploidentical Transplantation
Source: Transplant Cell Ther. Author manuscript; Available in PMC 2026 May 18. (PMC13181854; doi:10.1016/j.jtct.2025.03.020)
Supplement: 2 [file NIHMS2173896-supplement-2.docx]

# Supplementary Table 1: Patient Factors Associated with Melphalan cAUC

| **Patient or Transplant Factor** | **Overall** | **Melphalan cAUC**  **Median (range) in mg*hr/L** | **P value** |
| --- | --- | --- | --- |
| Overall | 85 | 6.81 (4.4-8.88) | - |
| Age  <10 years  ≥10 years | 42 (49.4%)  43 (50.6%) | 6.87 (4.4-8.76)  6.54 (5.62-8.88) | **0.03** |
| Body Mass Index (per CDC)  Not done (<2 years old)  Underweight  Healthy Weight  Overweight  Obese | 14 (16.5%)  6 (7.1%)  43 (50.6%)  8 (9.4%)  14 (16.5%) | 6.77 (4.4-8.76)  6.88 (6.52-8.82)  6.86 (6.06-8.88)  6.75 (6.2-7.66)  6.12 (5.62-7.24) | **0.02** |
| HCT Number  First  Second | 76 (89.4%)  9 (10.6%) | 6.8 (4.4-8.88)  6.88 (6.84-7.96) | **0.03** |
| Donor^1^  Mother  Father  Sibling (full- or half-)  Other (child) | 32 (37.6%)  20 (23.5%)  32 (37.6%)  1 (1.2%) | 6.89 (6.1-8.82)  6.86 (4.4-8.34)  6.57 (4.96-8.88)  7.26 | **<0.001** |

1: Patients with parental donors were younger (median age=5.9 years) compared to those with sibling donors (median age=15.8 years; p=0.003).

# Supplementary Table 2a. Patient Demographics and Transplant Characteristics Associated with Engraftment Syndrome

| **Patient or Transplant Factor** | **Events / Number** | **Day 100 ES**  **(95% CI)** | **P value** | **HR** | **P value** |
| --- | --- | --- | --- | --- | --- |
| Overall | 29/85 | 34.2% (24.2-44.2%) | - | - | **-** |
| Age  <10 years  ≥10 years | 18/42  11/43 | 43% (27.9-58.1%)  25.6% (12.5-38.7%) | 0.15 | 1.0  0.59 (.28-1.25) | 0.16 |
| Sex  Male  Female | 16/51  13/34 | 31.4% (18.7-44.1%)  38.4% (22.2-55.2%) | 0.66 | 1.0  1.18 (0.57-2.45) | 0.66 |
| Race & Ethnicity  White/Non-Hispanic  All Others | 5/18  24/67 | 27.8% (7-48.6%)  35.9% (24.3-47.5%) | 0.56 | 1.0  1.32 (0.5-3.47) | 0.57 |
| Body Mass Index (per CDC)  Not done (<2 years old)  Underweight  Healthy Weight  Overweight  Obese | 6/14  2/6  16/43  2/8  3/14 | 43.7% (17.2-70.2%)  33.3% (0.1-70.9%)  37.2% (22.7-51.7%)  25% (0.1-55%)  21.4% (0.1-43%) | 0.23 | 1.0  0.84 (0.17-4.15)  0.92 (0.36-2.34)  0.64 (0.13-3.18)  0.46 (0.11-1.83) | 0.82  0.83  0.86  0.59  0.29 |
| Disease  ALL  AML/MDS  Other | 15/48  9/24  5/13 | 31.3% (18.2-44.4%)  37.5% (18.1-56.9%)  38.5% (12-65%) | 0.84 | 1.0  1.22 (0.53-2.79)  1.27 (046-3.49) | 0.85  0.64  0.65 |
| MRD Status  Negative (by NGS or Flow)  Positive (by NGS or Flow)  Not done | 21/60  4/20  4/5 | 35.1% (22.9-47.3%)  20% (2.6-37.4%)  80% (44.9-99.9%) | **0.007** | 1.0  0.55 (0.19-1.6)  3.72 (1.27-10.9) | **0.02**  0.27  **0.02** |
| HCT Number  First  Second | 24/76  5/9 | 31.6% (21.2-42%)  55.6% (23.1-88.1%) | 0.13 | 1.0  2.03 (0.77-5.32) | 0.15 |
| Donor  Mother  Father  Sibling (full- or half-)  Other (child)* | 13/32  7/20  8/32  1/1 | 40.6% (23.5-57.7%)  35% (14-56%)  25% (9.9-40.1%)  100% (16.8-100%) | **0.02** | 1.0  0.87 (0.35-2.19)  0.59 (0.25-1.43)  - | 0.5  0.77  0.24  - |
| Donor Age  <30 years  ≥30 years | 13/40  16/45 | 32.5% (18-47%)  35.6% (21.7-49.5%) | 0.79 | 1.0  1.1 (0.53-2.29) | 0.8 |
| CMV Serostatus  R-/D-  R-/D+  R+/D+  R+/D- | 6/22  9/17  12/42  2/4 | 27.3% (8.7-45.9%)  52.9% (29.2-76.6%)  28.6% (14.9-42.3%)  50% (1-99%) | 0.39 | 1.0  2.05 (0.73-5.77)  1.11 (0.42-2.97)  1.92 (0.39-9.52) | 0.42  0.17  0.83  0.43 |
| rATG Exposure Optimized  No  Yes | 16/51  13/34 | 31.5% (18.8-44.2%)  38.8% (22.5-55.1%) | 0.4 | 1.0  1.36 (0.65-2.83) | 0.41 |
| CD34 Cell Dose, median  <18.7 x10^6/kg  ≥18.7 x10^6/kg | 18/42  11/43 | 42.9% (28-57.8%)  25.7% (12.6-38.8%) | 0.07 | 1.0  0.52 (0.24-1.09) | 0.08 |
| A/B T-Cell Dose, median  <7.5 x10^4/kg  7.5 x10^4/kg  >7.5 x10^4/kg | 12/23  5/29  12/33 | 52.2% (24.8-79.6%)  17.2% (3.5-30.9%)  36.5% (20-53%) | **0.04** | 1.0  0.28 (0.1-0.8)  0.63 (0.28-1.39) | 0.06  **0.02**  0.25 |
| Tocilizumab  No  Yes | 19/38  10/47 | 50.3% (36.9-69.1%)  21.3% (9.5-33.1%) | **0.008** | 1.0  0.38 (0.18-0.81) | **0.01** |
| Melphalan Predicted Exposure  ≤6.8 mg*hr/L  >6.8 mg*hr/L | 8/42  21/43 | 19.1% (7.1-31.1%)  48.8% (33.9-63.7%) | **0.005** | 1.0  2.94 (1.3-6.66) | **0.01** |

*Category excluded from Cox regression model.

# Supplementary Table 2b. Bivariate Analysis of Risk Factors Associated with Engraftment Syndrome Controlling for Melphalan Exposure

| **Variable** | **N Events /**  **N Total** | **HR estimate**  **(95% CI)** | **P value** | **HR estimate (95% CI) For High Melphalan Exposure^** | **P value** |
| --- | --- | --- | --- | --- | --- |
| MRD Status  Negative (by NGS or Flow)  Positive (by NGS or Flow)  Not done | 21/60  4/20  4/5 | 1.0  0.52 (0.18-1.51)  3.33 (1.13-9.84) | **0.03**  0.23  **0.03** | 2.9 (1.28-6.6) | **0.01** |
| CD34 Cell Dose, median  <18.7 x10^6/kg  ≥18.7 x10^6/kg | 18/42  11/43 | 1.0  0.47 (0.22-0.99) | **0.046** | 3.17 (1.4-7.2) | **0.006** |
| A/B T-Cell Dose, median  <7.5 x10^4/kg  7.5 x10^4/kg  ≥7.5 x10^4/kg | 12/23  5/29  12/33 | 1.0  0.32 (0.11-0.9)  0.66 (0.3-1.48) | 0.1  **0.03**  0.32 | 2.72 (1.2-6.17) | **0.02** |
| Tocilizumab  No  Yes | 19/38  10/47 | 1.0  0.44 (0.2-0.96) | **0.04** | 2.54 (1.11-5.82) | **0.03** |

^Comparator=Low Melphalan Exposure (≤6.8 mg*hr/L)

# Supplementary Table 3a. Patient Demographics and Transplant Characteristics Associated with Grade II-IV aGVHD

| **Patient or Transplant Factor** | **Events / Number** | **Day 100 Grade II-IV aGVHD (95% CI)** | **P value** | **HR** | **P value** |
| --- | --- | --- | --- | --- | --- |
| Overall | 20/85 | 24.8% (15.4-34.2%) | - | - | **-** |
| Age  <10 years  ≥10 years | 10/42  10/43 | 25% (11.5-38.5%)  24.8% (11.3-38.3%) | 0.9 | 1.0  0.95 (.039-2.27) | **0.9** |
| Sex  Male  Female | 12/51  8/34 | 24.9% (12.6-37.2%)  24.7% (9.8-39.6%) | 0.89 | 1.0  0.94 (0.38-2.3) | 0.89 |
| Race & Ethnicity  White/Non-Hispanic  All Others | 2/18  18/67 | 11.5% (0.1-26.4%)  28.4% (17.2-39.6%) | 0.15 | 1.0  2.78 (0.65-12) | 0.17 |
| Body Mass Index (per CDC)  Not done (<2 years old)  Underweight  Healthy Weight  Overweight  Obese | 1/14  2/6  12/43  1/8  4/14 | 7.7% (0.1-22.2%)  37.5% (0.1-79.2%)  29.9% (15.4-44.4%)  12.5% (0.1-35.4%)  28.6% (4.9-52.3%) | 0.44 | 1.0  5.83 (0.53-64.48)  4.64 (0.6-35.72)  1.75 (0.11-28.01)  4.26 (0.48-38.13) | 0.53  0.15  0.14  0.69  0.2 |
| Disease  ALL  AML/MDS  Other | 14/48  4/24  2/13 | 30.9% (17.4-44.4%)  18% (1.7-34.3%)  13.4% (0.1-33%) | 0.43 | 1.0  0.55 (0.18-1.68)  0.51 (0.12-2.23) | 0.44  0.3  0.37 |
| MRD Status  Negative (by NGS or Flow)  Positive (by NGS or Flow)  Not done | 16/60  2/20  2/5 | 28.3% (16.5-40.1%)  10% (0.1-23.1%)  40% (0.1-82.9%) | 0.18 | 1.0  0.35 (0.08-1.53)  1.94 (0.45-8.44) | 0.22  0.16  0.38 |
| HCT Number  First  Second | 16/76  4/9 | 22.5% (12.7-32.3%)  43.4% (10.9-75.9%) | 0.08 | 1.0  2.59 (0.87-7.77) | 0.09 |
| Donor  Mother  Father  Sibling (full- or half-)  Other (child)* | 11/32  2/20  6/32  1/1 | 37.2% (19.6-54.8%)  10% (0.1-23.1%)  19.3% (5.4-33.2%)  100% (16.8-100%) | **<0.001** | 1.0  0.24 (0.05-1.07)  0.42 (0.16-1.14)  - | 0.07  0.06  0.09  - |
| Donor Age  <30 years  ≥30 years | 9/40  11/45 | 22.6% (9.7-35.5%)  27.6% (13.5-41.7%) | 0.74 | 1.0  1.16 (0.48-2.8) | 0.75 |
| CMV Serostatus  R-/D-  R-/D+  R+/D+  R+/D- | 6/22  5/17  8/42  1/4 | 28.6% (9.2-48%)  30.7% (8.4-53%)  20.7% (7.8-33.6%)  25% (0.1-67.5%) | 0.87 | 1.0  1.1 (0.34-3.6)  0.71 (0.25-2.06)  0.88 (0.11-7.29) | 0.88  0.88  0.53  0.9 |
| rATG Exposure Optimized  No  Yes | 14/51  6/34 | 29.7% (16.6-42.8%)  17.6% (4.9-30.3%) | 0.34 | 1.0  0.63 (0.24-1.64) | 0.35 |
| CD34 Cell Dose, median  <18.7 x10^6/kg  ≥18.7 x10^6/kg | 7/42  13/43 | 17.4% (5.6-29.2%)  31.9% (17.6-46.2%) | 0.17 | 1.0  1.89 (.075-4.74) | 0.18 |
| A/B T-Cell Dose, median  <7.5 x10^4/kg  7.5 x10^4/kg  >7.5 x10^4/kg | 6/23  5/29  9/33 | 27.9% (8.9-46.9%)  18% (3.7-32.3%)  28.7% (12.8-44.6%) | 0.55 | 1.0  0.58 (0.18-1.9)  1.01 (0.36-2.85) | 0.56  0.37  0.98 |
| Tocilizumab  No  Yes | 11/38  9/47 | 30.8% (15.5-46.1%)  19.4% (8-30.8%) | 0.13 | 1.0  0.52 (0.21-1.25) | 0.15 |
| Melphalan Predicted Exposure  ≤6.8 mg*hr/L  >6.8 mg*hr/L | 4/42  16/43 | 10.1% (0.7-19.5%)  39.3% (24.2-54.4%) | **0.002** | 1.0  4.76 (1.59-14.25) | **0.005** |

*Category excluded from Cox regression model.

# Supplementary Table 3b. Bivariate Analysis of Risk Factors Associated with Grade II-IV aGVHD Controlling for Melphalan Exposure

| **Variable** | **N Events /**  **N Total** | **HR estimate**  **(95% CI)** | **P value** | **HR estimate (95% CI) For High Melphalan Exposure^** | **P value** |
| --- | --- | --- | --- | --- | --- |
| HCT Number  First  Second | 16/76  4/9 | 1.0  1.64 (0.54-5.02) | 0.39 | 4.76 (1.59-14.25) | **0.005** |
| Donor  Mother  Father  Sibling (full- or half-) | 11/32  2/20  6/32 | 1.0  0.25 (0.05-1.11)  0.78 (0.27-2.26) | 0.19  -  0.07  0.64 | 4.51 (1.5-13.6) | **0.007** |

^Comparator=Low Melphalan Exposure (≤6.8 mg*hr/L)

# Supplementary Table 4a. Patient Demographics and Transplant Characteristics Associated with Grade III-IV aGVHD

| **Patient or Transplant Factor** | **Events / Number** | **Day 100 Grade III-IV aGVHD (95% CI)** | **P value** | **HR** | **P value** |
| --- | --- | --- | --- | --- | --- |
| Overall | 14/85 | 17.1% (8.9-25.3%) | - | - | **-** |
| Age  <10 years  ≥10 years | 8/42  6/43 | 19.9% (7.6-32.2%)  14.3% (3.7-24.9%) | 0.52 | 1.0  0.71 (0.25-2.05) | 0.53 |
| Sex  Male  Female | 9/51  5/34 | 18.5% (7.5-29.5%)  15.1% (2.9-27.3%) | 0.68 | 1.0  0.8 (0.27-2.37) | 0.68 |
| Race & Ethnicity  White/Non-Hispanic  All Others | 2/18  12/67 | 11.5% (0.1-26.4%)  18.5% (9.1-27.9%) | 0.44 | 1.0  1.78 (0.4-7.95) | 0.45 |
| Body Mass Index (per CDC)  Not done (<2 years old)  Underweight  Healthy Weight  Overweight  Obese | 1/14  2/6  7/43  1/8  3/14 | 7.7% (0.1-22.2%)  37.5% (0.1-79.2%)  16.5% (5.3-27.7%)  12.5% (0.1-35.4%)  21.4% (0.1-43%) | 0.65 | 1.0  5.48 (0.5-60.54)  2.53 (0.31-20.58)  1.76 (0.11-28.15)  3.0 (0.31-28.9) | 0.69  0.17  0.39  0.69  0.34 |
| Disease  ALL  AML/MDS  Other | 9/48  3/24  2/13 | 19.5% (8.1-30.9%)  13.7% (0.1-28.4%)  15.4% (0.1-35%) | 0.8 | 1.0  0.65 (0.18-2.4)  0.83 (0.18-3.82) | 0.81  0.52  0.81 |
| MRD Status  Negative (by NGS or Flow)  Positive (by NGS or Flow)*  Not done | 13/60  0/20  1/5 | 22.5% (11.7-33.3%)  0% (0-19%)  20% (0.1-55.1%) | 0.09 | 1.0  -  1.03 (0.14-7.91) | 0.99  -  0.97 |
| HCT Number  First  Second | 10/76  4/9 | 13.8% (5.8-21.8%)  44.4% (11.9-76.9%) | **0.009** | 1.0  4.16 (1.3-13.29) | **0.03** |
| Donor  Mother  Father  Sibling (full- or half-)  Other (child)* | 9/32  2/20  2/32  1/1 | 30.3% (13.6-47%)  10% (0.1-23.1%)  6.2% (0.1-14.6%)  100% (16.8-100%) | **<0.001** | 1.0  0.31 (0.07-1.43)  0.18 (0.04-0.83)  - | **0.03**  0.13  **0.03**  **-** |
| Donor Age  <30 years  ≥30 years | 5/40  9/45 | 12.5% (2.3-22.7%)  21.8% (9.1-34.5%) | 0.32 | 1.0  1.73 (0.58-5.18) | 0.33 |
| CMV Serostatus  R-/D-  R-/D+  R+/D+  R+/D-* | 4/22  5/17  5/42  0/4 | 19% (2.1-35.9%)  30.3% (8-52.6%)  12% (2.2-21.8%)  0% (0-54.6%) | 0.37 | 1.0  1.69 (0.45-6.31)  0.68 (0.18-2.53)  - | 0.55  0.43  0.57  - |
| rATG Exposure Optimized  No  Yes | 9/51  5/34 | 18.8% (7.6-30%)  14.7% (2.7-26.7%) | 0.72 | 1.0  0.82 (0.28-2.45) | 0.73 |
| CD34 Cell Dose, median  <18.7 x10^6/kg  ≥18.7 x10^6/kg | 5/42  9/43 | 12.1% (2.1-22.1%)  21.9% (9.2-34.6%) | 0.31 | 1.0  1.75 (0.59-5.23) | 0.32 |
| A/B T-Cell Dose, median  <7.5 x10^4/kg  7.5 x10^4/kg  >7.5 x10^4/kg | 4/23  2/29  8/33 | 18.5% (2-35%)  7.2% (0.1-16.8%)  24.9% (10-39.8%) | 0.17 | 1.0  0.35 (0.07-1.93)  1.42 (0.43-4.72) | 0.21  0.23  0.59 |
| Tocilizumab  No  Yes | 9/38  5/47 | 25% (10.7-39.3%)  10.6% (1.8-19.4%) | 0.058 | 1.0  0.36 (0.12-1.09) | 0.07 |
| Melphalan Predicted Exposure  ≤6.8 mg*hr/L  >6.8 mg*hr/L | 1/42  13/43 | 2.5% (0.1-7.4%)  31.5% (17.2-45.8%) | **<0.001** | 1.0  15.1 (1.97-115.54) | **0.009** |

*Category excluded from Cox regression model.

# Supplementary Table 4b. Bivariate Analysis of Risk Factors Associated with Grade III-IV aGVHD Controlling for Melphalan Exposure

| **Variable** | **N Events /**  **N Total** | **HR estimate**  **(95% CI)** | **P value** | **HR estimate (95% CI) For High Melphalan Exposure^** | **P value** |
| --- | --- | --- | --- | --- | --- |
| HCT Number  First  Second | 10/76  4/9 | 1.0  2.29 (0.71-7.39) | 0.17 | 12.94 (1.66-101.1) | **0.015** |
| Donor  Mother  Father  Sibling (full- or half-) | 9/32  2/20  2/32 | 1.0  0.33 (0.07-1.51)  0.43 (0.09-2.14) | 0.27  -  0.15  0.31 | 14.13 (1.84-108.79) | **0.01** |
| Tocilizumab  No  Yes | 9/38  5/47 | 1.0  0.47 (0.16-1.4) | 0.17 | 13.62 (1.77-104.77) | **0.011** |

^Comparator=Low Melphalan Exposure (≤6.8 mg*hr/L)

# Supplementary Table 5a. Patient Demographics and Transplant Characteristics Associated with cGVHD

| Patient or Transplant Factor | Events / Number | 3-year cGVHD  (95% CI) | P value | HR | P value |
| --- | --- | --- | --- | --- | --- |
| Overall | 11/85 | 17.5% (8.1-26.9%) | - | - | **-** |
| Age  <10 years  ≥10 years | 6/42  5/43 | 18.8% (5.1-32.5%)  16% (3.1-28.9%) | 0.61 | 1.0  0.73 (0.23-2.4) | 0.61 |
| Sex  Male  Female | 8/51  3/34 | 20.3% (7.8-32.8%)  12.3% (0.1-25.6%) | 0.47 | 1.0  0.62 (0.16-2.33) | 0.48 |
| Race & Ethnicity  White/Non-Hispanic  All Others | 1/18  10/67 | 7.7% (0.1-22.2%)  19.9% (8.7-31.1%) | 0.35 | 1.0  2.57 (0.33-20.07) | 0.37 |
| Body Mass Index (per CDC)  Not done (<2 years old)*  Underweight  Healthy Weight  Overweight  Obese | 0/14  1/6  7/43  1/8  2/14 | 0% (0-25.2%)  20% (0.1-55.1%)  23% (7.9-38.1%)  14.3% (0.1-40.2%)  19.2% (0.1-43.1%) | 0.58 | -  1.0  0.82 (0.1-6.66)  0.56 (0.04-8.98)  0.67 (0.06-7.36) | 0.99  -  0.85  0.68  0.74 |
| Disease  ALL  AML/MDS  Other | 7/48  2/18  2/11 | 19.8% (6.5-33.1%)  11.6% (0.1-26.9%)  20% (0.1-44.7%) | 0.71 | 1.0  0.55 (0.11-2.64)  1.11 (0.23-5.36) | 0.72  0.45  0.9 |
| MRD Status  Negative (by NGS or Flow)  Positive (by NGS or Flow)*  Not done | 10/60  0/20  1/5 | 22% (9.8-34.2%)  0% (0-19%)  25% (0.1-67.5%) | 0.18 | 1.0  -  1.21 (0.16-9.45) | 0.98  -  0.86 |
| HCT Number  First  Second | 9/76  2/7 | 15.9% (6.3-25.5%)  31.4% (0.1-67.9%) | 0.3 | 1.0  2.22 (0.48-10.28) | 0.31 |
| Donor  Mother  Father  Sibling (full- or half-)  Other (child)* | 6/32  2/20  2/32  1/1 | 28.9% (9.1-48.7%)  14.3% (0.1-32.7%)  8% (0.1-18.6%)  100% (16.8-100%) | **<0.001** | 1.0  0.49 (0.1-2.42)  0.23 (0.05-1.16)  - | 0.19  0.38  0.08  - |
| Donor Age  <30 years  ≥30 years | 5/40  6/45 | 15.1% (2.8-27.4%)  20.4% (5.7-35.1%) | 0.56 | 1.0  1.43 (0.44-4.67) | 0.56 |
| CMV Serostatus  R-/D-  R-/D+  R+/D+  R+/D-* | 4/22  2/17  5/42  0/4 | 22.9% (2.9-42.9%)  16.1% (0.1-36.5%)  16.5% (3.2-29.8%)  0% (0-54.6%) | 0.83 | 1.0  0.7 (0.13-3.82)  0.73 (0.2-2.72)  - | 0.97  0.68  0.64  - |
| rATG Optimized  No  Yes | 8/51  3/34 | 20.7% (7.8-33.6%)  12% (0.1-24.9%) | 0.37 | 1.0  0.55 (0.15-2.08) | 0.38 |
| CD34 Cell Dose, median  <18.7 x10^6/kg  ≥18.7 x10^6/kg | 3/42  8/43 | 9.3% (0.1-19.5%)  25.2% (9.9-40.5%) | 0.14 | 1.0  2.64 (0.7-9.96) | 0.15 |
| A/B T-Cell Dose, median  <7.5 x10^4/kg  7.5 x10^4/kg  >7.5 x10^4/kg | 5/23  1/29  5/33 | 30.4% (8.1-52.7%)  5.3% (0.1-15.3%)  21% (5.9-36.1%) | 0.12 | 1.0  0.14 (0.02-1.18)  0.65 (0.19-2.25) | 0.19  0.07  0.5 |
| Tocilizumab  No  Yes | 8/38  3/47 | 33% (14.6-51.4%)  7.9% (0.1-16.5%) | **0.02** | 1.0  0.22 (0.06-0.84) | **0.03** |
| Melphalan Predicted Exposure  ≤6.8 mg*hr/L  >6.8 mg*hr/L | 2/42  9/43 | 7.4% (0.1-17.2%)  27.2% (11.9-42.5%) | **0.03** | 1.0  4.84 (1.05-22.39) | **0.04** |

*Category excluded from Cox regression model.

# Supplementary Table 5b. Bivariate Analysis of Risk Factors Associated with cGVHD Controlling for Melphalan Exposure

| **Variable** | **N Events /**  **N Total** | **HR estimate**  **(95% CI)** | **P value** | **HR estimate (95% CI) For High Melphalan Exposure^** | **P value** |
| --- | --- | --- | --- | --- | --- |
| Donor  Mother  Father  Sibling (full- or half-) | 6/32  2/20  2/32 | 1.0  0.59 (0.12-2.98)  0.41 (0.07-2.4) | 0.57  -  0.53  0.32 | 2.95 (0.53-16.42) | 0.22 |
| A/B T-Cell Dose, median  <7.5 x10^4/kg  7.5 x10^4/kg  >7.5 x10^4/kg | 5/23  1/29  5/33 | 1.0  0.2 (0.02-1.73)  0.85 (0.24-3) | 0.34  0.14  0.8 | 3.94 (0.83-18.77) | 0.09 |
| Tocilizumab  No  Yes | 8/38  3/47 | 1.0  0.27 (0.07-1.04) | 0.06 | 3.91 (0.84-18.34) | 0.08 |

^Comparator=Low Melphalan Exposure (≤6.8 mg*hr/L)

# Supplementary Table 6a. Cause of NRM by Melphalan Exposure Group

| **Cause of Mortality** | **Low Melphalan Exposure**  **(≤6.8 mg*hr/L)** | **High Melphalan Exposure**  **(>6.8 mg*hr/L)** |
| --- | --- | --- |
| Total | 2 | 3 |
| Organ Failure  Respiratory Failure, etiology unknown  Fludarabine neurotoxicity | 1  0  1 | 2  2  0 |
| Chronic GVHD | 1 | 1 |

# Supplementary Table 6b. Patient Demographics and Transplant Characteristics Associated with NRM

| **Patient or Transplant Factor** | **Events / Number** | **3-year NRM**  **(95% CI)** | **P value** |
| --- | --- | --- | --- |
| Overall | 5/85 | 8.7% (1.1-16.3%) | - |
| Age  <10 years  ≥10 years | 2/42  3/43 | 7.5% (0.1-17.9%)  9.7% (0.1-20.3%) | 0.64 |
| Sex  Male  Female | 3/51  2/34 | 9.7% (0.1-20.5%)  7.2% (0.1-16.8%) | 0.98 |
| Race & Ethnicity  White/Non-Hispanic  All Others | 1/18  4/67 | 7.1% (0.1-20.6%)  8.9% (0.3-17.5%) | 0.96 |
| Body Mass Index (per CDC)  Not done (<2 years old)  Underweight  Healthy Weight  Overweight  Obese | 0/14  0/6  3/43  0/8  2/14 | 0% (0-25.2%)  0% (0-44.3%)  9.8% (0.1-20.6%)  0% (0-37.2%)  18.5% (0.1-41.8%) | 0.52 |
| Disease  ALL  AML/MDS  Other | 3/48  0/24  2/13 | 8.3% (0.1-17.5%)  0% (0-16.3%)  20.5% (0.1-46.2%) | 0.25 |
| MRD Status  Negative (by NGS or Flow)  Positive (by NGS or Flow)  Not done | 3/60  1/20  1/5 | 6.3% (0.1-13.4%)  7.7% (0.1-22.2%)  25% (0.1-70.7%) | 0.53 |
| HCT Number  First  Second | 4/76  1/9 | 8.6% (0.2-17%)  11.1% (0.1-31.7%) | 0.62 |
| Donor  Mother  Father  Sibling (full- or half-)  Other (child)* | 2/32  1/20  2/32  0/1 | 10.5% (0.1-25%)  6.7% (0.1-19.2%)  8% (0.1-18.8%)  0% (0-83.3%) | 0.99 |
| Donor Age  <30 years  ≥30 years | 2/40  3/45 | 6.4% (0.1-15%)  10.9% (0.1-23.2%) | 0.55 |
| CMV Serostatus  R-/D-  R-/D+  R+/D+  R+/D- | 0/22  2/17  2/42  1/4 | 0% (0-17.6%)  14.1% (0.1-32.3%)  8.8% (0.1-21%)  33.3% (0.1-86.6%) | 0.21 |
| rATG Exposure Optimized  No  Yes | 4/51  1/34 | 11.6% (0.4-22.8%)  3.8% (0.1-11.2%) | 0.41 |
| CD34 Cell Dose, median  <18.7 x10^6/kg  ≥18.7 x10^6/kg | 2/42  3/43 | 5.4% (0.1-12.7%)  12.4% (0.1-25.9%) | 0.63 |
| A/B T-Cell Dose, median  <7.5 x10^4/kg  7.5 x10^4/kg  >7.5 x10^4/kg | 1/23  2/29  2/33 | 6.2% (0.1-18.2%)  4% (0.1-11.6%)  8.9% (0.1-21.2%) | 0.94 |
| Tocilizumab  No  Yes | 3/38  2/47 | 12.5% (0.2-24.8%)  4.8% (0.1-11.3%) | 0.49 |
| Melphalan Predicted Exposure  ≤6.8 mg*hr/L  >6.8 mg*hr/L | 2/42  3/43 | 7.4% (0.1-17.2%)  9.2% (0.1-19.4%) | 0.82 |

# Supplementary Table 7. Patient Demographics and Transplant Characteristics Associated with Relapse

| **Patient or Transplant Factor** | **Events / Number** | **3-year Relapse**  **(95% CI)** | **P value** |
| --- | --- | --- | --- |
| Overall | 15/85 | 21.8% (12-31.6%) | - |
| Age  <10 years  ≥10 years | 8/42  7/43 | 22.9% (9-36.8%)  20.9% (6.8-35%) | 0.81 |
| Sex  Male  Female | 9/51  6/34 | 22.7% (9.6-35.8%)  19.7% (5.4-34%) | 0.99 |
| Race & Ethnicity  White/Non-Hispanic  All Others | 5/18  10/67 | 36.3% (10.4-62.2%)  18.5% (8.3-28.7%) | 0.19 |
| Body Mass Index (per CDC)  Not done (<2 years old)  Underweight  Healthy Weight  Overweight  Obese | 3/14  1/6  7/43  2/8  2/14 | 23.1% (0.2-46%)  20% (0.1-55.1%)  21.2% (7.3-35.1%)  27.1% (0.1-59.4%)  20.9% (0.1-47.9%) | 0.99 |
| Disease  ALL  AML/MDS  Other | 9/48  4/24  2/13 | 23.5% (9.8-37.2%)  21.4% (2.8-40%)  17.5% (0.1-39.6%) | 0.95 |
| MRD Status  Negative (by NGS or Flow)  Positive (by NGS or Flow)  Not done | 7/60  8/20  0/5 | 14.2% (4.4-24%)  50.8% (25.3-76.3%)  0% (0-48.9%) | **0.004** |
| HCT Number  First  Second | 13/76  2/9 | 21.1% (10.7-31.5%)  25% (0.1-55%) | 0.84 |
| Donor  Mother  Father  Sibling (full- or half-)  Other (child) | 7/32  4/20  4/32  0/1 | 25.9% (9.4-42.4%)  30.7% (5.4-56%)  15.6% (1.3-29.9%)  0% (0-83.3%) | 0.58 |
| Donor Age  <30 years  ≥30 years | 6/40  9/45 | 17.2% (4.5-29.9%)  26.9% (11.8-42%) | 0.28 |
| CMV Serostatus  R-/D-  R-/D+  R+/D+  R+/D- | 2/22  2/17  10/42  1/4 | 10.5% (0.1-24.2%)  14.3% (0.1-32.7%)  30.2% (14.3-46.1%)  25% (0.1-67.5%) | 0.36 |
| rATG Exposure Optimized  No  Yes | 10/51  5/34 | 23.4% (10.7-36.1%)  18.8% (3.3-34.3%) | 0.71 |
| CD34 Cell Dose, median  <18.7 x10^6/kg  ≥18.7 x10^6/kg | 8/42  7/43 | 23.8% (9.3-38.3%)  19% (6.3-31.7%) | 0.84 |
| A/B T-Cell Dose, median  <7.5 x10^4/kg  7.5 x10^4/kg  >7.5 x10^4/kg | 2/23  7/29  6/33 | 11.1% (0.1-25.6%)  33.2% (11.8-54.6%)  21.4% (6.1-36.7%) | 0.39 |
| Tocilizumab  No  Yes | 4/38  11/47 | 14.3% (1.4-27.2%)  27.9% (13.6-42.2%) | 0.27 |
| Melphalan Predicted Exposure  ≤6.8 mg*hr/L  >6.8 mg*hr/L | 9/42  6/43 | 27.6% (11.9-43.3%)  16.8% (4.5-29.1%) | 0.31 |

# Supplementary Table 8. Patient Demographics and Transplant Characteristics Associated with RFS

| **Patient or Transplant Factor** | **Events / Number** | **3-year RFS**  **(95% CI)** | **P value** |
| --- | --- | --- | --- |
| Overall | 20/85 | 71.4% (60.6-82.2%) | - |
| Age  <10 years  ≥10 years | 10/42  10/43 | 71.4% (56.1-86.7%)  71.4% (56.3-86.5%) | 0.98 |
| Sex  Male  Female | 12/51  8/34 | 69.8% (55.3-84.3%)  74.5% (59.1-89.7%) | 0.98 |
| Race & Ethnicity  White/Non-Hispanic  All Others | 6/18  12/56 | 59.2% (33.7-84.7%)  74.7% (63.1-86.3%) | 0.25 |
| Body Mass Index (per CDC)  Not done (<2 years old)^1^  Underweight  Health Weight  Overweight  Obese | 3/14  1/6  10/43  2/8  4/14 | 76.9% (54-99.8%)  80% (44.9-99.9%)  71.1% (55.8-86.4%)  72.9% (40.6-99.9%)  64.5% (0.1-99.9%) | 0.99 |
| Disease  ALL  AML/MDS  Other | 12/48  4/24  4/13 | 70.1% (55.6-84.6%)  78.6% (60-97.2%)  65.6% (38.2-93%) | 0.74 |
| MRD Status  Negative (by NGS or Flow)  Positive (by NGS or Flow)  Not done | 10/60  9/20  1/5 | 80.4% (69.4-91.4%)  45.4% (20.9-69.9%)  75% (32.5-99.9%) | **0.02** |
| HCT Number  First  Second | 17/76  3/9 | 72.1% (60.5-83.7%)  66.7% (35.9-97.5%) | 0.66 |
| Donor  Mother  Father  Sibling (full- or half-)  Other (child) | 9/32  5/20  6/32  0/1 | 66.3% (48.1-84.5%)  64.6% (39.3-89.9%)  77.7% (61.8-93.6%)  0% (0-83.3%) | 0.62 |
| Donor Age  <30 years  ≥30 years | 8/40  12/45 | 77.5% (63.6-91.4%)  65.1% (48.8-81.4%) | 0.21 |
| CMV Serostatus  R-/D-  R-/D+  R+/D+  R+/D- | 2/22  4/17  12/42  2/4 | 89.5% (75.8-99.9%)  73.7% (51.6-95.8%)  63.6% (46.7-80.5%)  50% (1-99%) | 0.21 |
| rATG Optimized  No  Yes | 14/51  6/34 | 67.7% (53.6-81.8%)  78.1% (62-94.2%) | 0.46 |
| CD34 Cell Dose, median  <18.7 x10^6/kg  ≥18.7 x10^6/kg | 10/42  10/43 | 72% (57.3-86.7%)  71% (55.3-86.7%) | 0.95 |
| A/B T-Cell Dose, median  <7.5 x10^4/kg  7.5 x10^4/kg  >7.5 x10^4/kg | 3/23  9/29  8/33 | 83.3% (66.1-99.9%)  61.2% (40.4-82%)  71.6% (54.7-88.5%) | 0.33 |
| Tocilizumab  No  Yes | 5/27  12/43 | 75.9% (60.2-91.6%)  68.6% (54.1-83.1%) | 0.54 |
| Melphalan Predicted Exposure  ≤6.8 mg*hr/L  >6.8 mg*hr/L | 11/42  9/43 | 66.8% (50.3-83.3%)  75.5% (61.6-89.4%) | 0.44 |

# Supplementary Table 9a. Patient Demographics and Transplant Characteristics Associated with GRFS

| **Patient or Transplant Factor** | **Events / Number** | **3-year GRFS**  **(95% CI)** | **P value** | **HR (for 1-GRFS)** | **P value** |
| --- | --- | --- | --- | --- | --- |
| Overall | 33/85 | 55.6% (44-67.2%) | - | - | **-** |
| Age  <10 years  ≥10 years | 17/42  16/43 | 54.2% (37.9-70.5%)  56.7% (40.2-73.2%) | 0.75 | 1.0  0.9 (0.45-1.78) | 0.75 |
| Sex  Male  Female | 19/51  14/34 | 56% (40.7-71.3%)  55.4% (37.8-73%) | 0.82 | 1.0  1.08 (0.54-2.16) | 0.82 |
| Race & Ethnicity  White/Non-Hispanic  All Others | 8/18  25/67 | 46.5% (20.4-72.6%)  58.1% (45.4-70.8%) | 0.74 | 1.0  0.87 (0.39-1.93) | 0.74 |
| Body Mass Index (per CDC)  Not done (<2 years old)^1^  Underweight  Healthy Weight  Overweight  Obese | 4/14  3/6  17/43  3/8  6/14 | 69.2% (44.1-94.3%)  41.7% (0.1-85.2%)  53.6% (37.1-70.1%)  60% (24.3-95.7%)  50.3% (19.9-80.7%) | 0.7 | 1.0  2.89 (0.68-12.99)  1.66 (0.56-4.93)  1.3 (0.29-5.79)  1.7 (0.48-6.04) | 0.72  0.17  0.36  0.74  0.41 |
| Disease  ALL  AML/MDS  Other | 21/48  7/24  5/13 | 49.8% (34.1-65.5%)  64.3% (42.7-85.9%)  59.2% (31.6-86.8%) | 0.61 | 1.0  0.65 (0.28-1.54)  0.84 (0.32-2.23) | 0.62  0.33  0.73 |
| MRD Status  Negative (by NGS or Flow)  Positive (by NGS or Flow)  Not done | 23/60  9/20  1/5 | 57.2% (43.7-70.7%)  45.4% (20.9-69.9%)  80% (44.9-99.9%) | 0.78 | 1.0  1.07 (0.49-2.31)  0.52 (0.07-3.83) | 0.79  0.87  0.52 |
| HCT Number  First  Second | 27/76  6/9 | 58.6% (46.4-70.8%)  33.3% (2.5-64.1%) | 0.052 | 1.0  2.34 (0.97-5.68) | 0.06 |
| Donor  Mother  Father  Sibling (full- or half-)  Other (child) | 17/32  7/20  8/32  1/1 | 40% (21.6-58.4%)  52.7%% (26-79.4%)  71.7% (54.8-88.6%)  0% (0.1-83.3%) | **<0.001** | 1.0  0.53 (0.35-1.51)  0.3 (0.13-0.69)  - | **0.02**  0.16  **0.005**  **-** |
| Donor Age  <30 years  ≥30 years | 13/40  20/45 | 64.9% (49.2-80.6%)  45.4% (28.7-62.1%) | 0.08 | 1.0  1.85 (0.92-3.74) | 0.08 |
| CMV Serostatus  R-/D-  R-/D+  R+/D+  R+/D- | 7/22  7/17  17/42  2/4 | 64.3% (42.7-85.9%)  55.7% (30.8-80.6%)  51.7% (34.6-68.8%)  50% (1-99%) | 0.91 | 1.0  1.37 (0.48-3.92)  1.36 (0.56-3.27)  1.38 (0.29-6.66) | 0.91  0.55  0.5  0.69 |
| rATG Optimized  No  Yes | 22/51  11/34 | 51.4% (36.7-66.1%)  62.5% (44.1-80.9%) | 0.48 | 1.0  0.77 (0.37-1.59) | 0.48 |
| CD34 Cell Dose, median  <18.7 x10^6/kg  ≥18.7 x10^6/kg | 14/42  19/43 | 62% (46.1-77.9%)  49.8% (33.5-66.1%) | 0.2 | 1.0  1.56 (0.78-3.12) | 0.21 |
| A/B T-Cell Dose, median  <7.5 x10^4/kg  7.5 x10^4/kg  ≥7.5 x10^4/kg | 7/23  12/29  14/33 | 64% (42.2-85.8%)  49.5% (28.1-70.9%)  53.6% (35.6-71.6%) | 0.74 | 1.0  1.19 (0.47-3.04)  1.42 (0.57-3.51) | 0.75  0.71  0.45 |
| Tocilizumab  No  Yes | 14/38  19/47 | 56.2% (38.4-74%)  55.1% (39.6-70.6%) | 0.52 | 1.0  0.8 (0.4-1.59) | 0.52 |
| Melphalan Predicted Exposure  ≤6.8 mg*hr/L  >6.8 mg*hr/L | 12/42  21/43 | 64.6% (48.1-81.1%)  46.4% (30.5-62.3%) | **0.02** | 1.0  2.23 (1.09-4.53) | **0.03** |

# Supplementary Table 9b. Bivariate Analysis of Risk Factors Associated with 1-GRFS Controlling for Melphalan Exposure

| **Variable** | **N Events /**  **N Total** | **HR estimate**  **(95% CI)** | **P value** | **HR estimate (95% CI) For High Melphalan Exposure^** | **P value** |
| --- | --- | --- | --- | --- | --- |
| HCT Number  First  Second | 27/76  6/9 | 1.0  1.83 (0.73-4.57) | 0.19 | 2.02 (0.97-4.21) | 0.06 |
| Donor  Mother  Father  Sibling (full- or half-) | 17/32  7/20  8/32 | 1.0  0.56 (0.23-1.36)  0.36 (0.15-0.89) | 0.07  0.2  **0.03** | 1.53 (0.71-3.31) | 0.28 |
| Donor Age  <30 years  ≥30 years | 13/40  20/45 | 1.0  1.54 (0.74-3.18) | 0.25 | 1.98 (0.95-4.14) | 0.07 |

^Comparator=Low Melphalan Exposure (≤6.8 mg*hr/L)

# Supplementary Table 10. Summary of Associations of Melphalan Exposure (Low vs. Medium vs. High) with Clinical Outcomes

|  |  | **Melphalan Low Exposure**  **(<6.7 mg*hr/L)^1^** | |  | **Melphalan Medium Exposure**  **(6.7-6.84 mg*hr/L)^2^** | |  | **Melphalan High Exposure (>6.85 mg*hr/L)^3^** | | **P value** |
| --- | --- | --- | --- | --- | --- | --- | --- | --- | --- | --- |
| **Outcome** | **Overall** | **Events** | **Cumulative Incidence**  **(95% CI)** |  | **Events** | **Cumulative Incidence**  **(95% CI)** |  | **Events** | **Cumulative Incidence**  **(95% CI)** |  |
| **N** |  | **33** |  |  | **13** |  |  | **39** |  |  |
| Engraftment Syndrome (Day 100) | 34.2%  (24.2-44.2%) | 7 | 21.3%  (7.2-35.4%) |  | 3 | 23.1%  (0.2-46%) |  | 19 | 48.7%  (33-64.4%) | **0.043** |
| Grade II-IV aGVHD (Day 100) | 24.8%  (15.4-34.2%) | 4 | 12.9%  (1.1-24.7%) |  | 2 | 15.4%  (0.1-35%) |  | 14 | 37.3%  (21.8-52.8%) | **0.046** |
| Grade III-IV aGVHD (Day 100) | 17.1%  (8.9-25.3%) | 1 | 3.1%  (0.1-9.2%) |  | 2 | 15.4%  (0.1-35%) |  | 11 | 29%  (14.5-43.5%) | **0.02** |
| cGVHD (3-year) | 19.2%  (8.6-29.8%) | 2 | 9.1%  (0.1-21.1%) |  | 1 | 16.7%  (0.1-46.5%) |  | 8 | 24.6%  (9.7-39.5%) | **0**.22 |
| NRM (3-year) | 8.7%  (1.1-16.3%) | 2 | 9.5%  (0.1-22.2%) |  | 1 | 10%  (0.1-28.6%) |  | 2 | 8.1%  (0.1-17.7%) | 0.73 |
| Relapse (3-year) | 21.8%  (12-31.6%) | 9 | 23.1%  (5.1-41.1%) |  | 0 | 0%  (0-32.1%) |  | 6 | 17.3%  (4.8-29.8%) | 0.14 |
| RFS (3-year) | 71.4%  (60.6-82.2%) | 11 | 60.5%  (42.1-78.9%) |  | 1 | 90%  (71.4-99.9%) |  | 8 | 76.9%  (62.8-91%) | 0.26 |
| GRFS (3-year) | 55.6%  (44-67.2%) | 12 | 57.6%  (39-76.2%) |  | 2 | 84.6%  (66.8-99.9%) |  | 19 | 48.1%  (31.8-64.4%) | 0.19 |

aGVHD = acute graft-versus-host disease; cGVHD = chronic graft-versus-host disease; NRM = non-relapse mortality; MRD = minimal residual disease; DFS = disease-free-survival; GRFS = GVHD-free, relapse-free survival.

1: Median exposure of 6.32 mg*hr/L (range, 4.4-6.68)

2: Median exposure of 6.8 mg*hr/L (range, 6-7-6.84)

3: Median exposure of 7.14 mg*hr/L (range, 6.86-8.88)

# Supplementary Table 11. Impacts of Age, Creatinine Clearance, and Obesity on Predicted Exposure of Melphalan in Hypothetical Male Patients

| **Hypothetical**  **Male**  **Patient** | **Age**  **(years)** | **CrCl**  **(ml/min/**  **1.73m^2^)** | **Height**  **(cm)** | **Weight /**  **A-IBW**  **(kg)** | **BSA /**  **A-BSA**  **(m2)** | **BSA-Dose**  **(Adjusted prn)**  **(mg)** | **Predicted cAUC**  **(mg*hr/L)** | **Model-Based Dose for Predicted cAUC of 6.8 mg*hr/L**  **(mg)** | **% Difference from BSA-Dose** |
| --- | --- | --- | --- | --- | --- | --- | --- | --- | --- |
| 1 | 0.75 | >120 | 72.5 | 9 / - | - **(c)** | 21* | 4.86 | 29.4 | +40% |
| 2 | 10 | >120 | 140 | 32.5 / - | 1.12 / - | 78.4 | 6.84 | 78 | -0.5% |
| 3 | 17.9 | >120 | 180 | 76.3 / - | 1.95 / - | 136.5 | 6.36 | 146 | +7% |
| 4 | 17.9 | >120 | 180 | 76.5 / 68.8 **(a)** | 1.96 / 1.85 | 129.5 | 6.02 | 146.2 | +12.9% |
| 5 | 17.9 | 90 | 180 | 76.3 / - | 1.95 / - | 136.5 | 7.42 | 125.3 | -8.2% |
| 6 | 17.9 | >120 | 180 | 90 / 74.2 **(b)** | 2.12 / 1.93 | 135.1 | 5.88 | 156.5 | +15.8% |
| 7 | 18.1 | >120 | 180 | 90 | 2.12 / - **(d)** | 148.4 | 6.46 | 156.5 | +5.5% |

Note: Female patients have differences in the calculations of CrCl, ideal body weight, and fat-free mass, so their results would look slightly different than above.

**(a)** Using Traub formula, IBW for a 180 cm 17.9 yo male is 63.7 kg and 120% of IBW is 76.4 kg.

**(b)** Using Devine formula, IBW for a 180 cm 18.1 yo male is 75.1 kg and 120% of IBW is 90.1 kg.

**(c)** Patients <10 kg are traditionally dosed at 2.33 mg/kg using the “rule of 30” (70 mg/m^2^ divided by 30) and not BSA.

**(d)** There is no standard practice regarding whether melphalan dosing should be capped at 140 mg for a patient >2.0 m2.
