# Supplementary material for: High Melphalan Exposure Increases the Risk of Graft-Versus-Host Disease in Pediatric Patients Undergoing Alpha-Beta T-Cell Depleted Haploidentical Transplantation
Source: Transplant Cell Ther. Author manuscript; Available in PMC 2026 May 18. (PMC13181854; doi:10.1016/j.jtct.2025.03.020)
Supplement: 3 [file NIHMS2173896-supplement-3.docx]

# Supplementary Figure 1


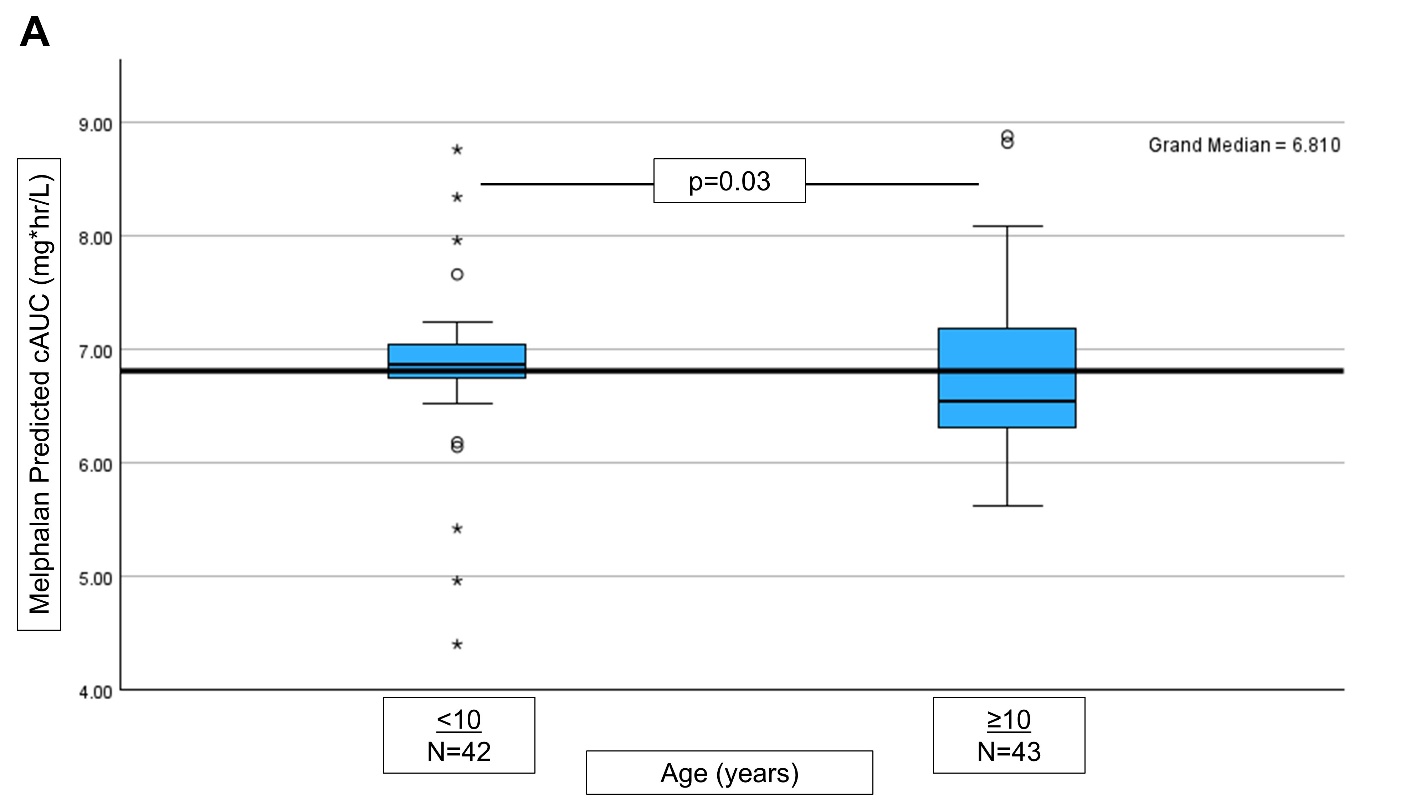


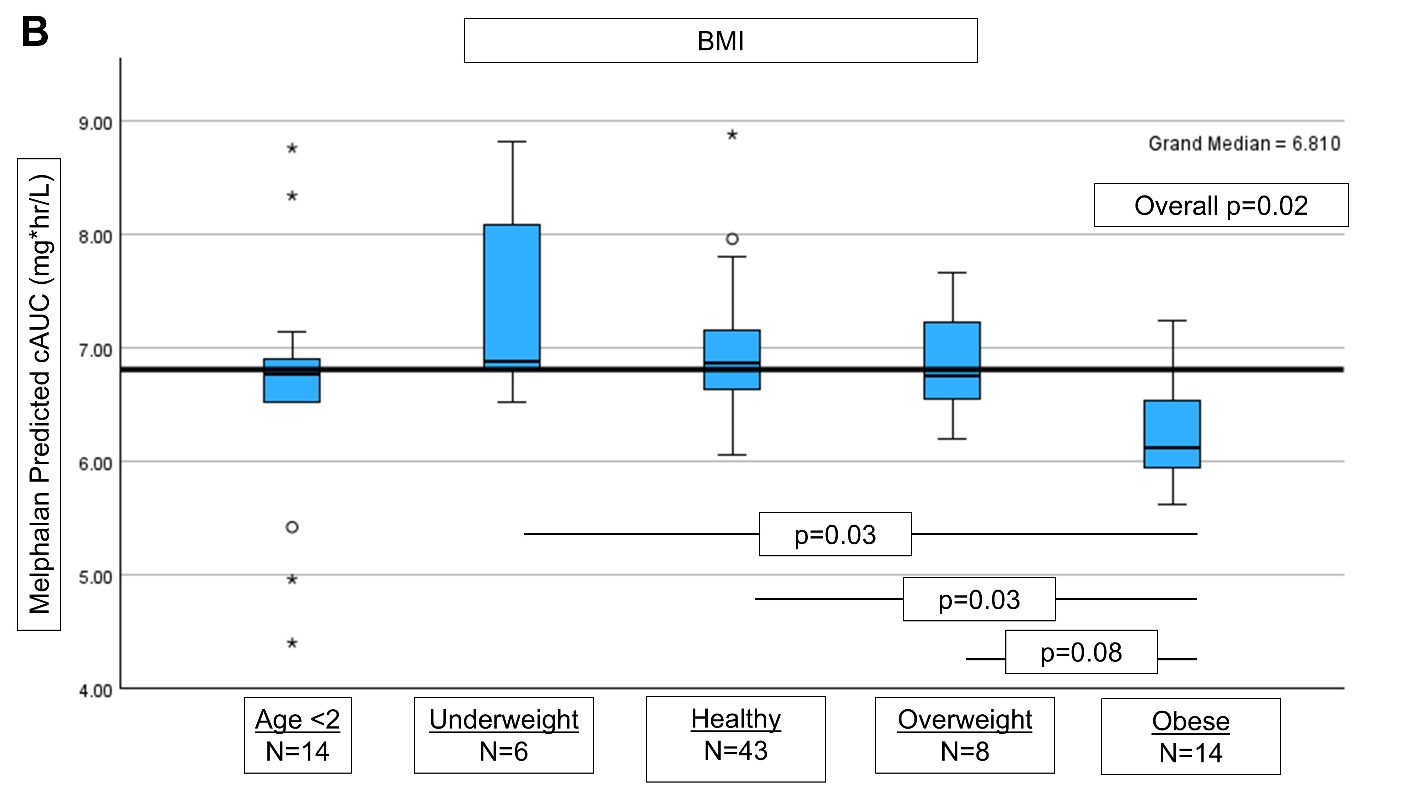


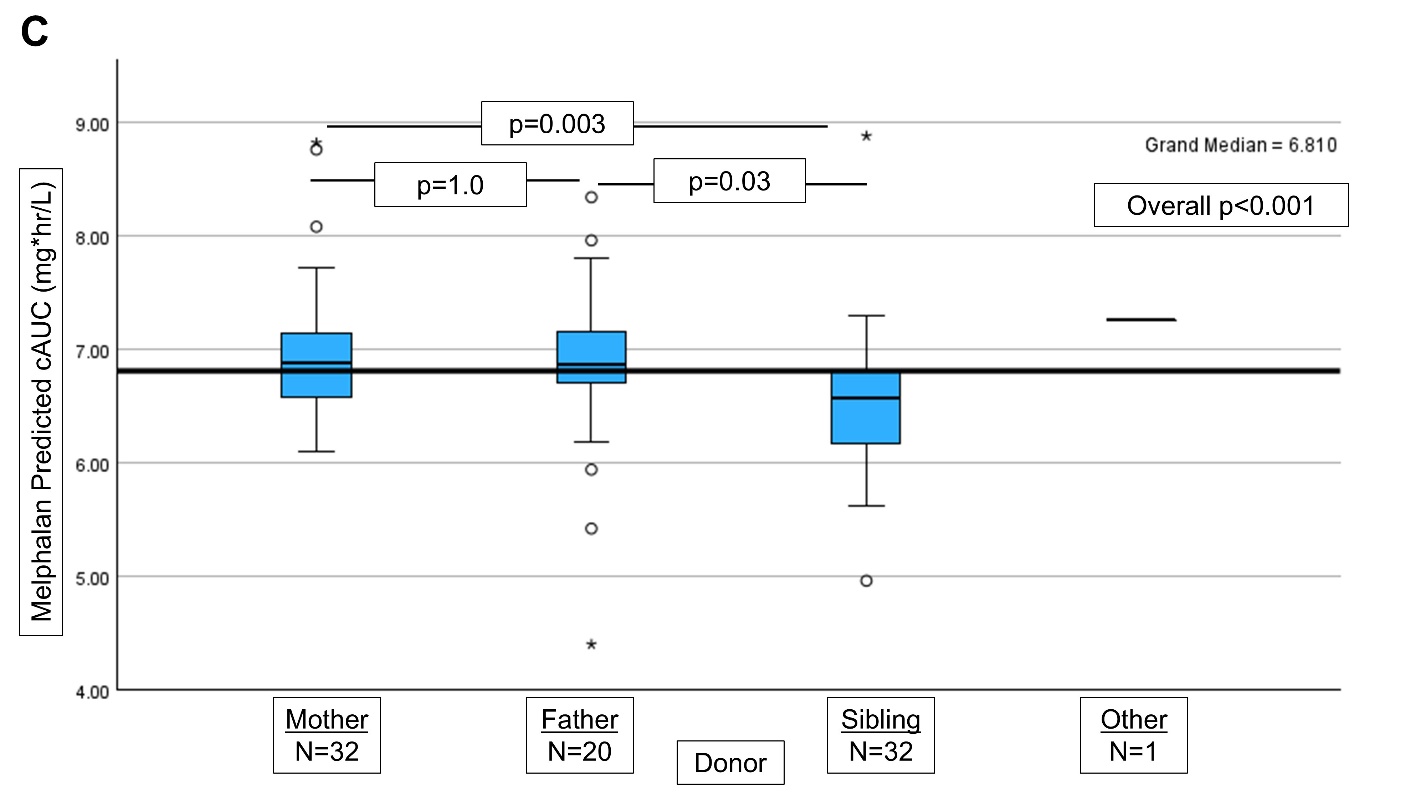


# Supplementary Figure 2


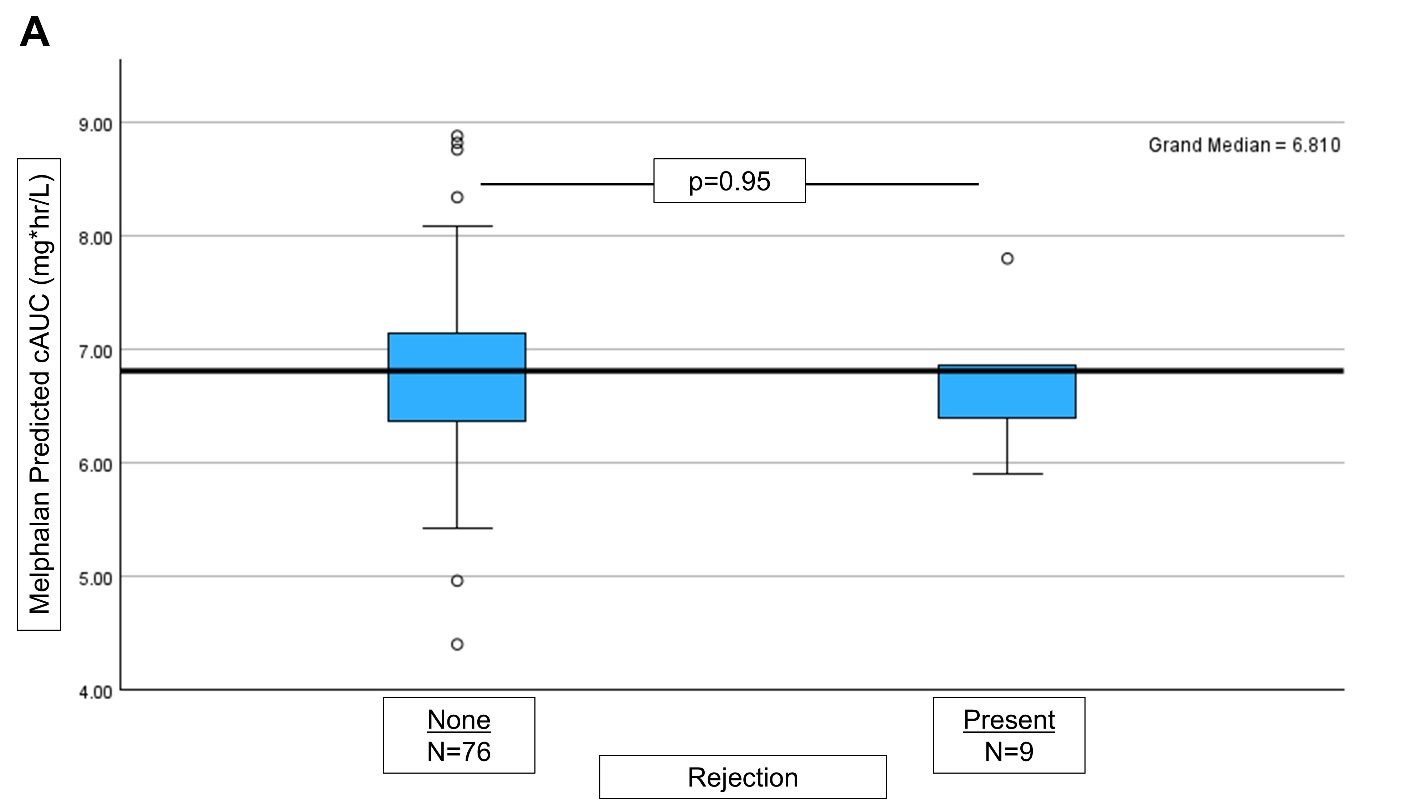


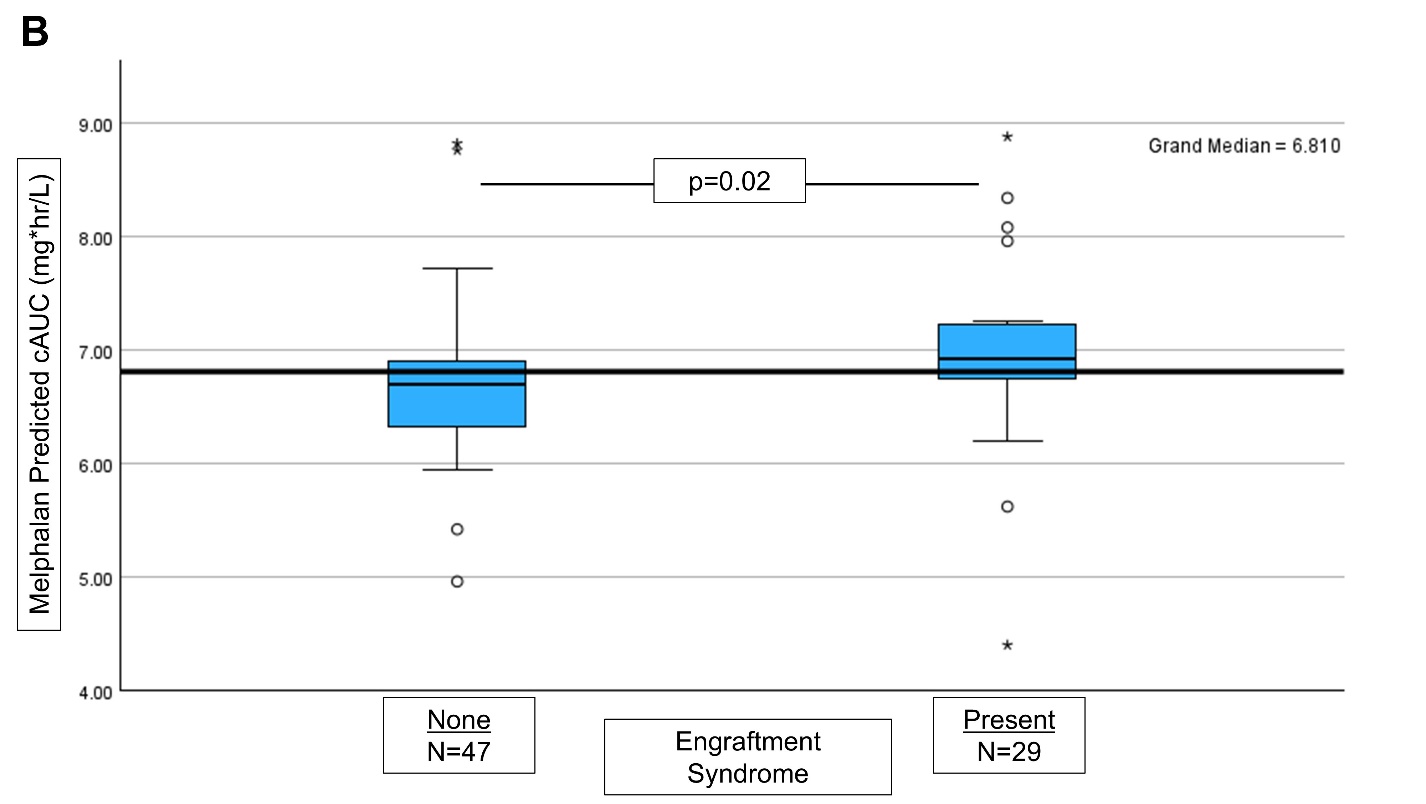


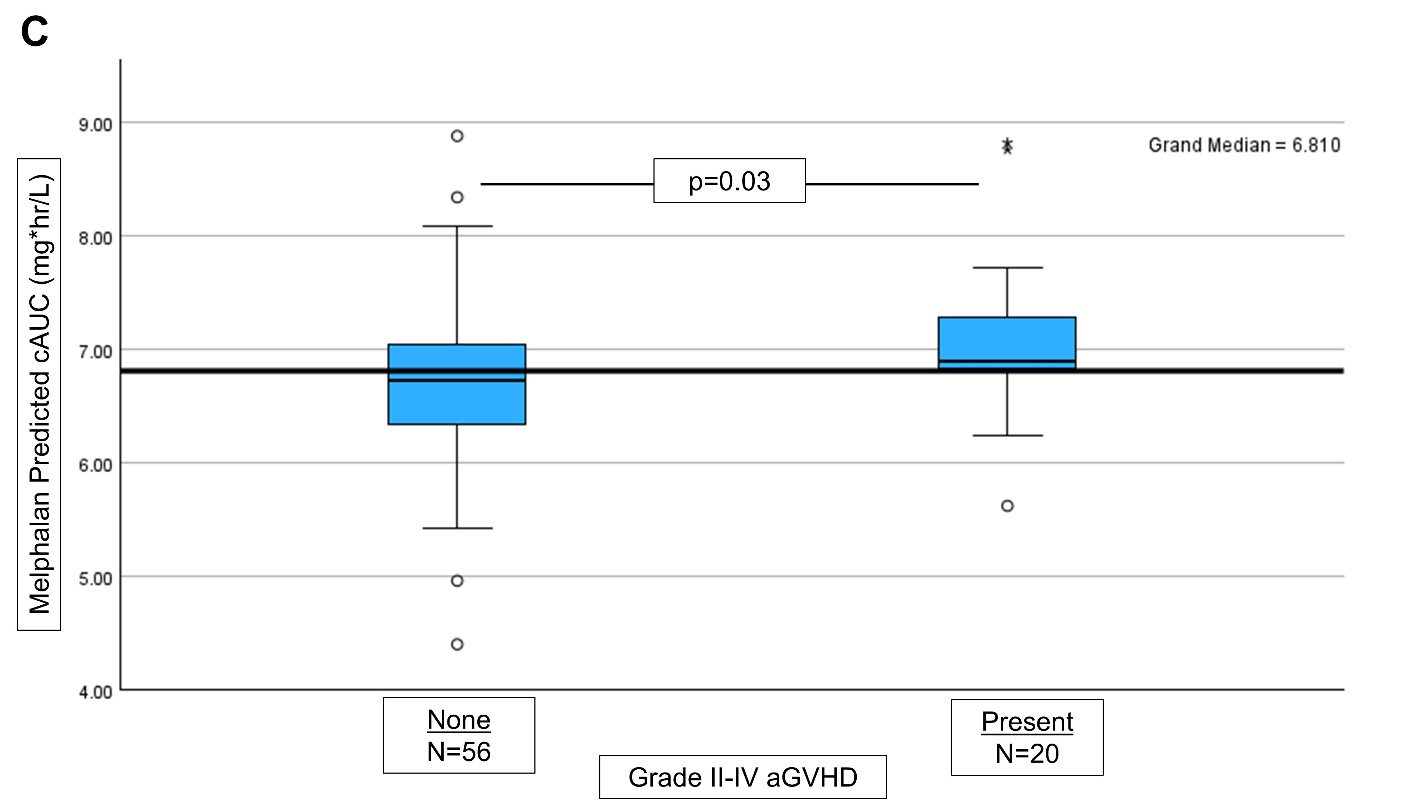


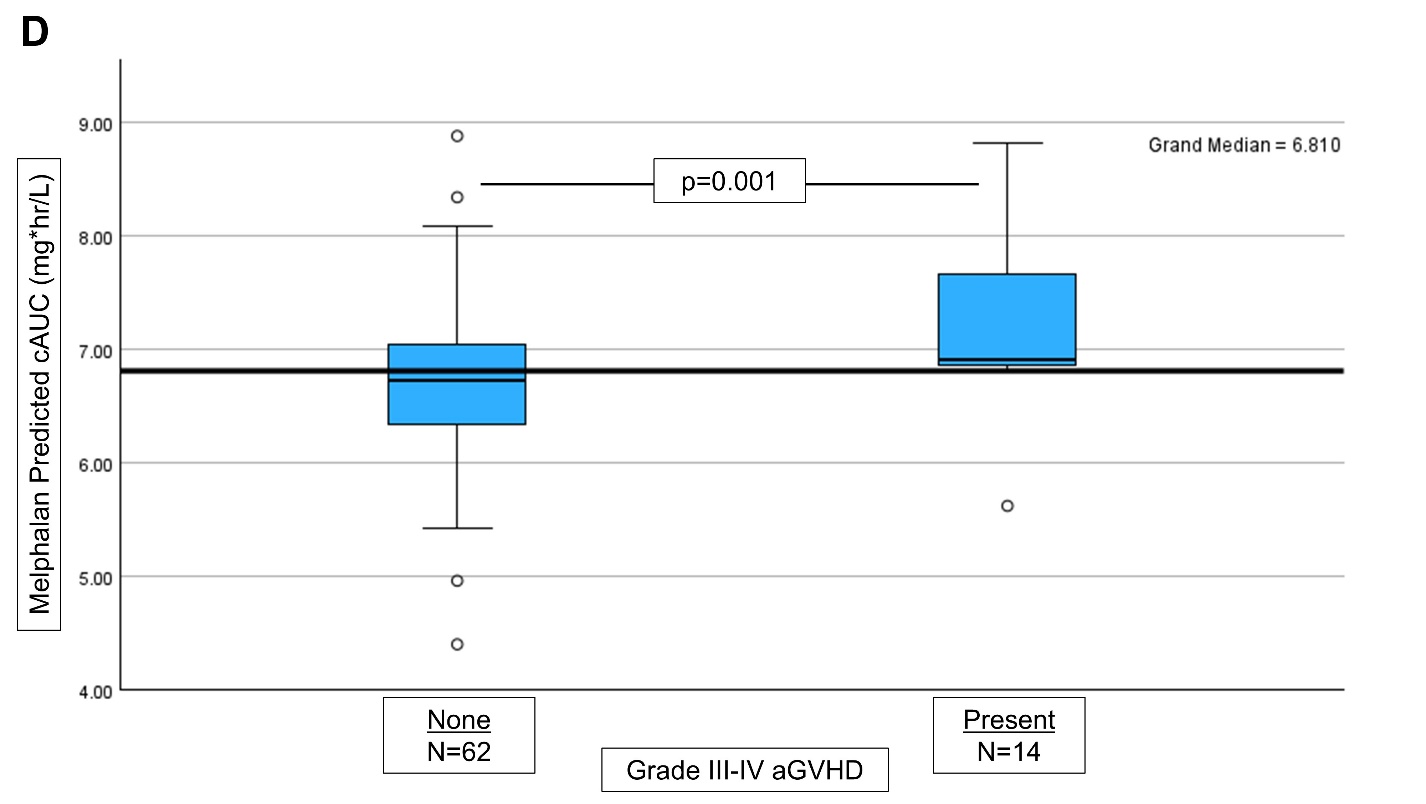


# Supplementary Figure 3


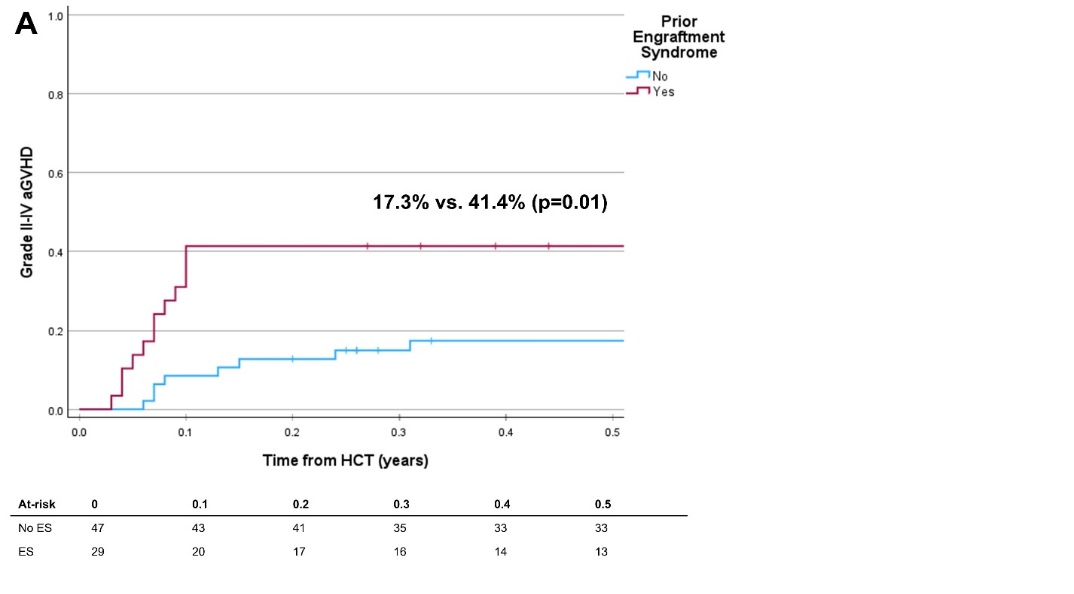
**Grade II-IV aGVHD by Prior ES Grade III-IV aGVHD by Prior ES**


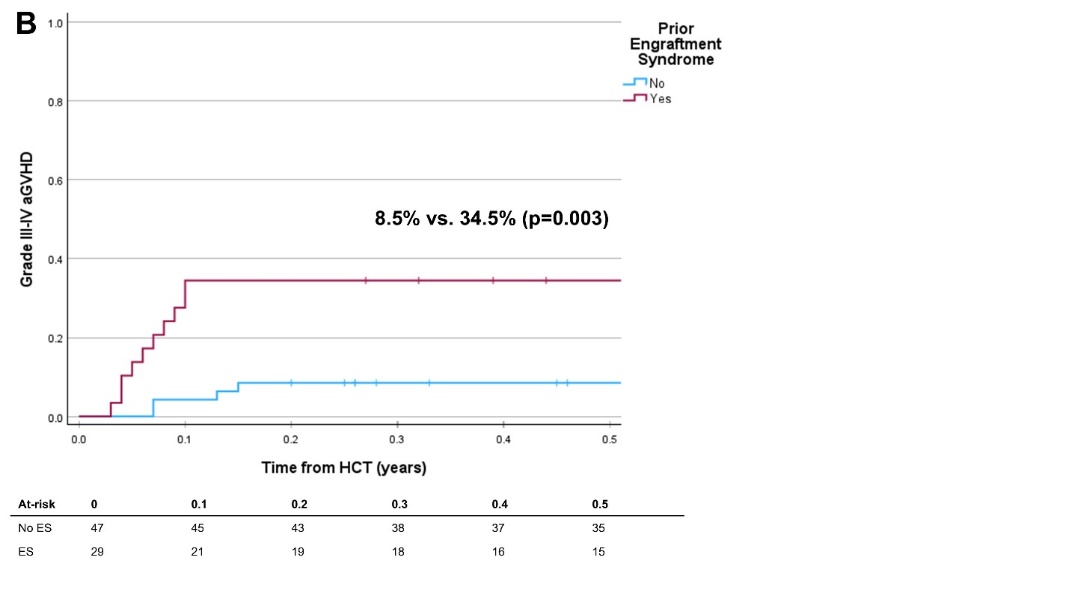


**cGVHD by Prior Grade II-IV aGVHD cGVHD by Prior Grade III-IV aGVHD**


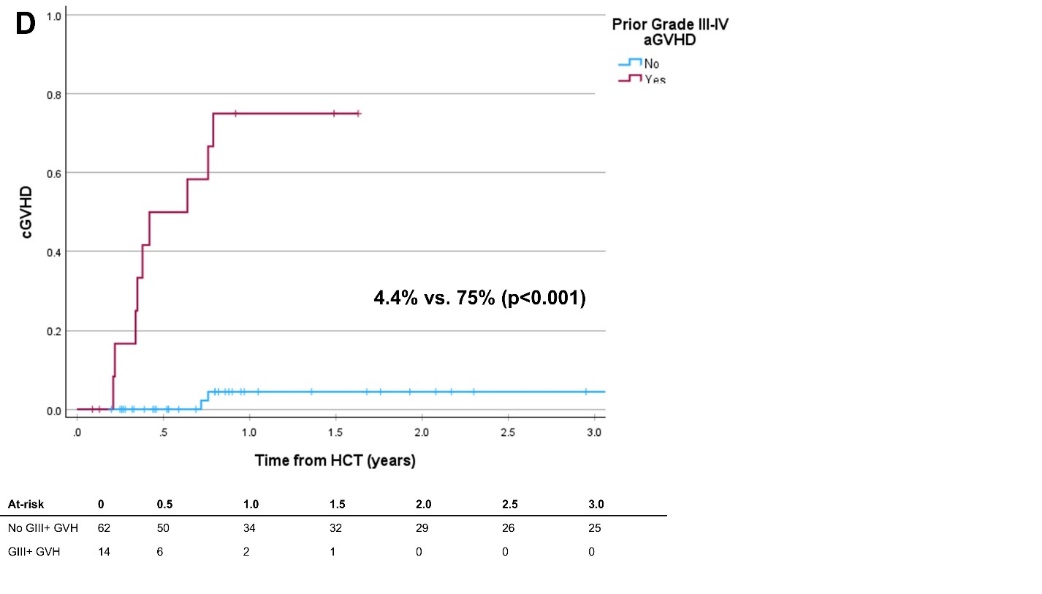

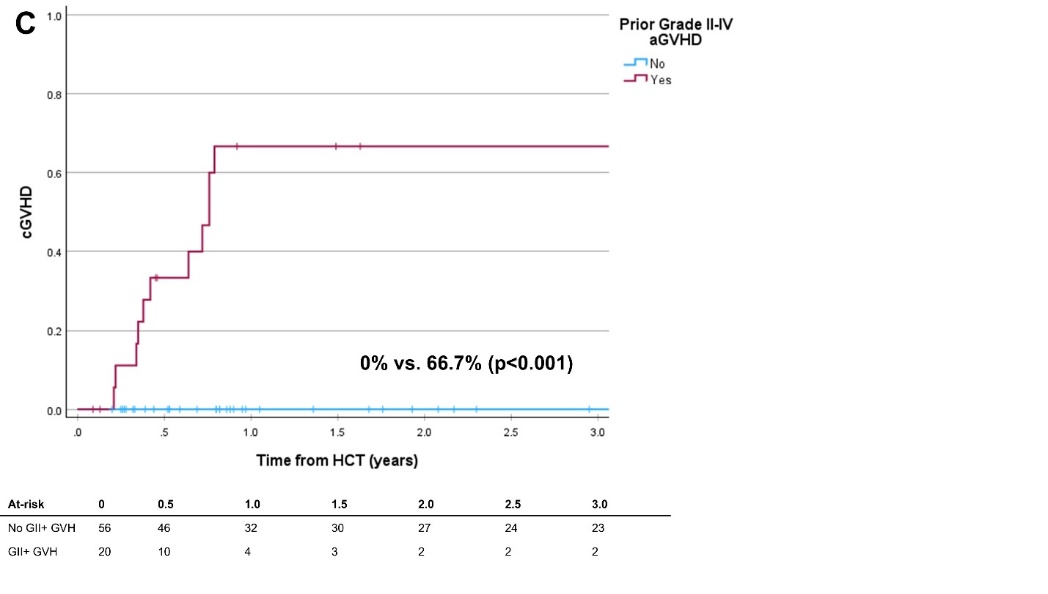


**
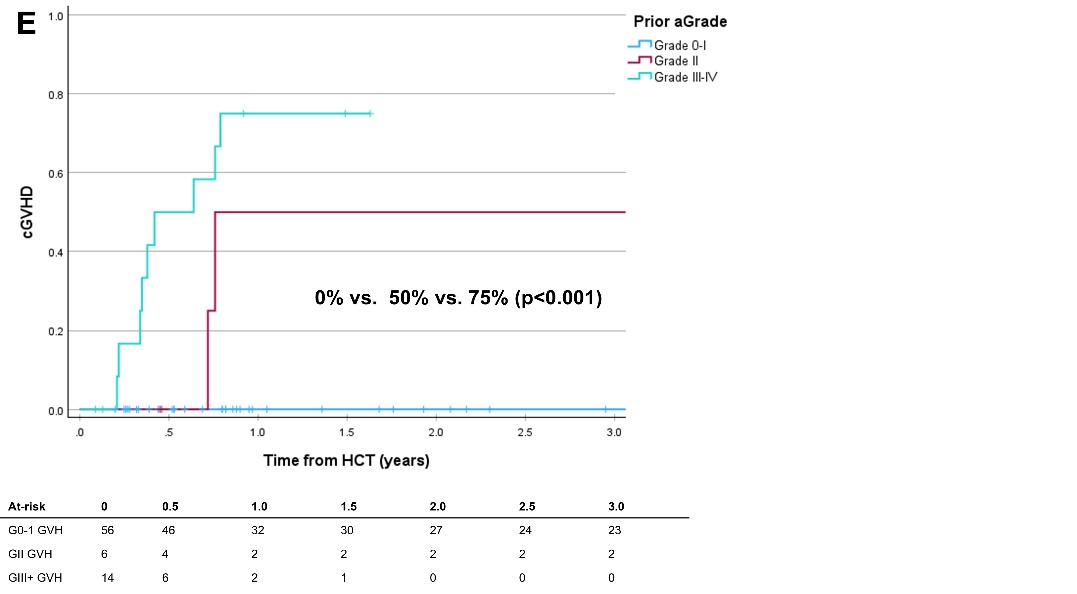

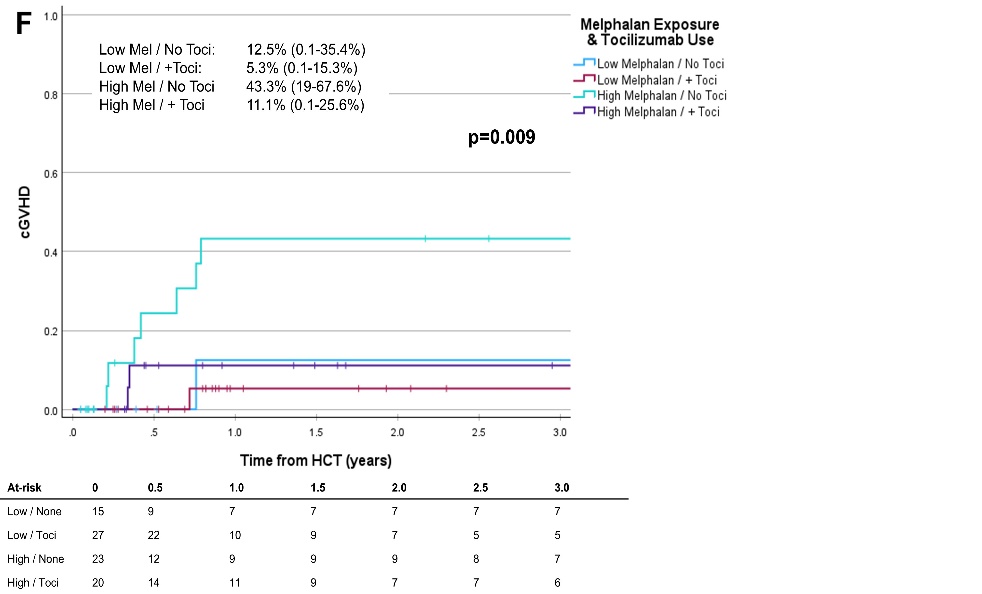
cGVHD by Prior aGVHD Grade cGVHD by Melphalan Exposure & Toci**

# Supplementary Figure 4

**
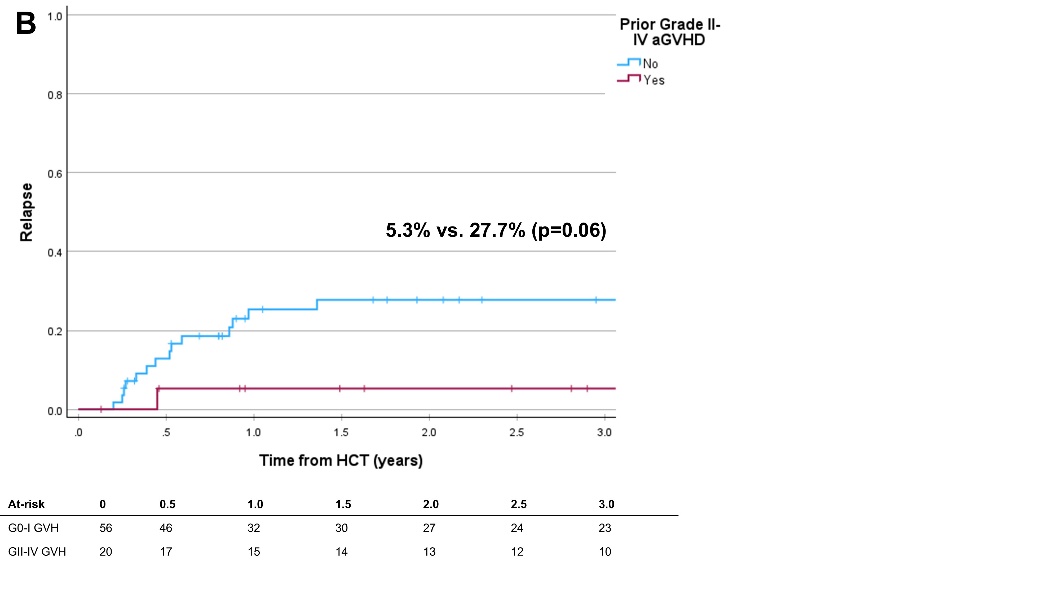
NRM by Prior Grade II-IV aGVHD Relapse
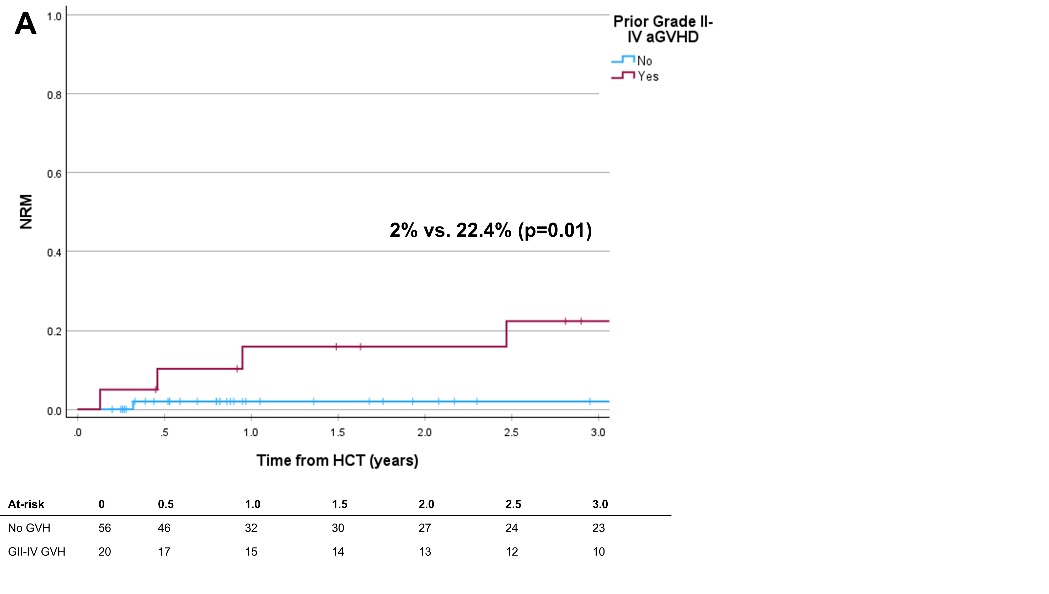
 by Prior Grade II-IV aGVHD**

**
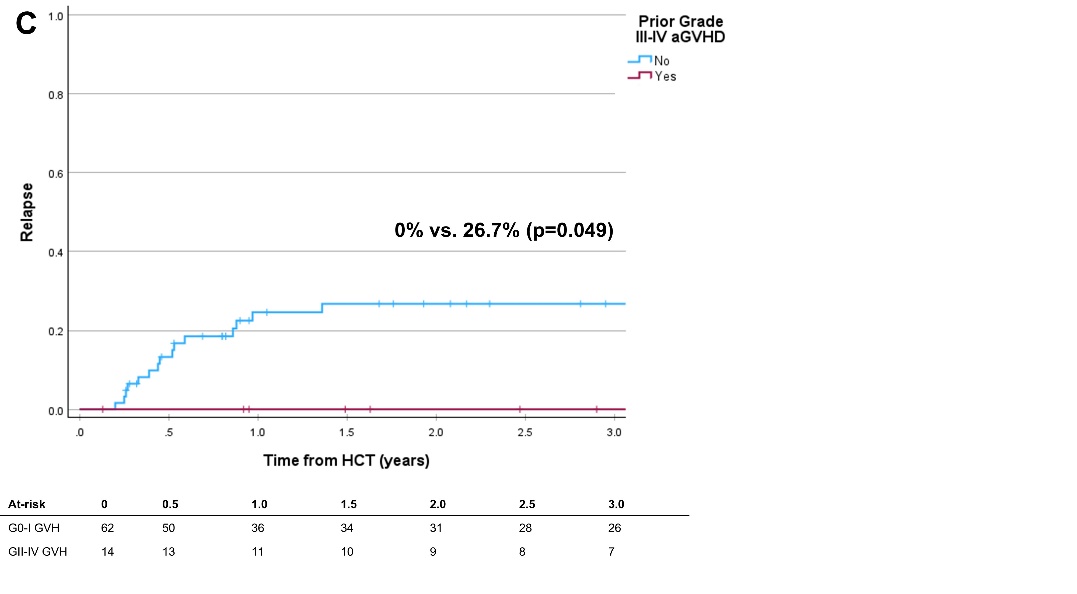
Relapse by Prior Grade III-IV aGVHD**

#
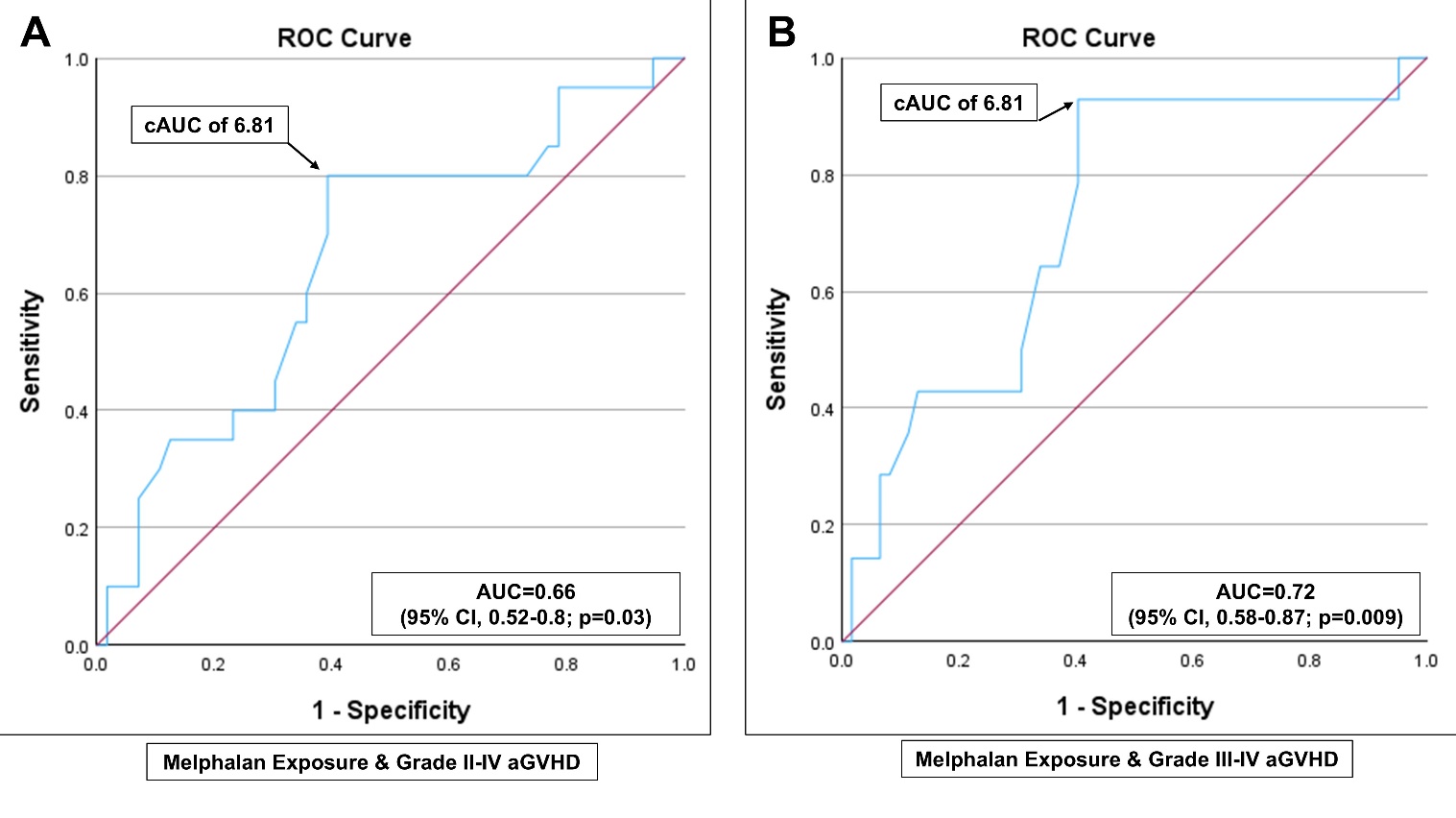
Supplementary Figure 5

# Supplementary Figure 6

**Engraftment Syndrome Grade II-IV Acute GVHD**


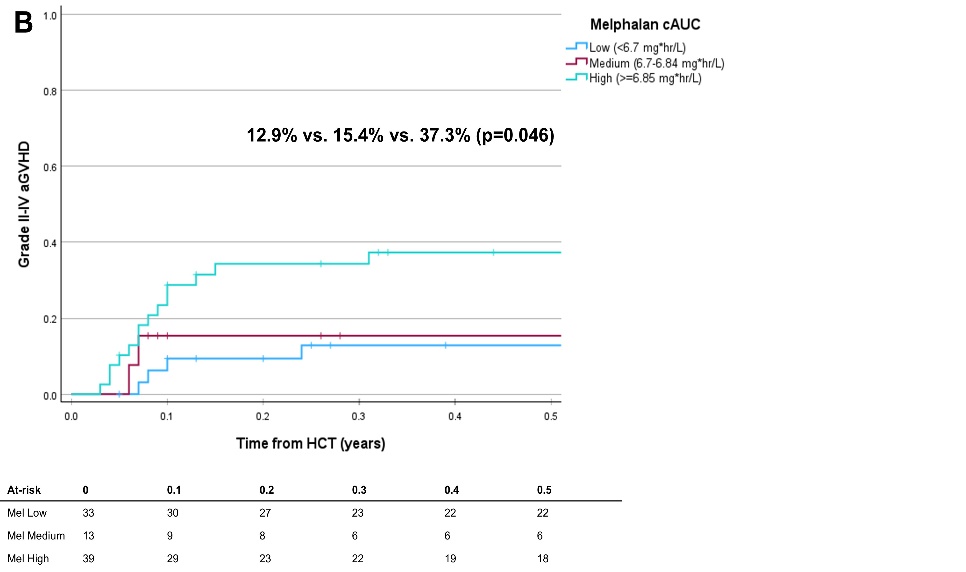

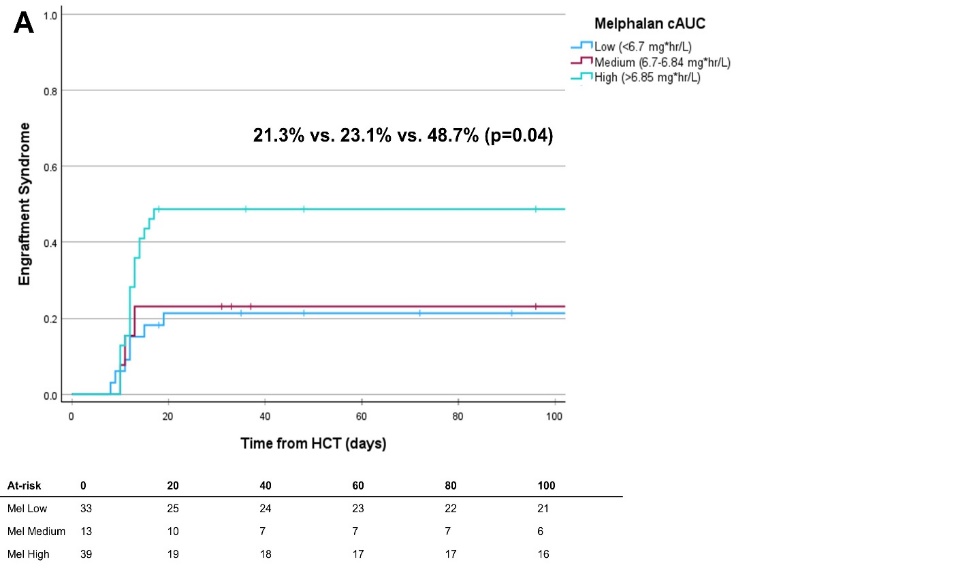


**
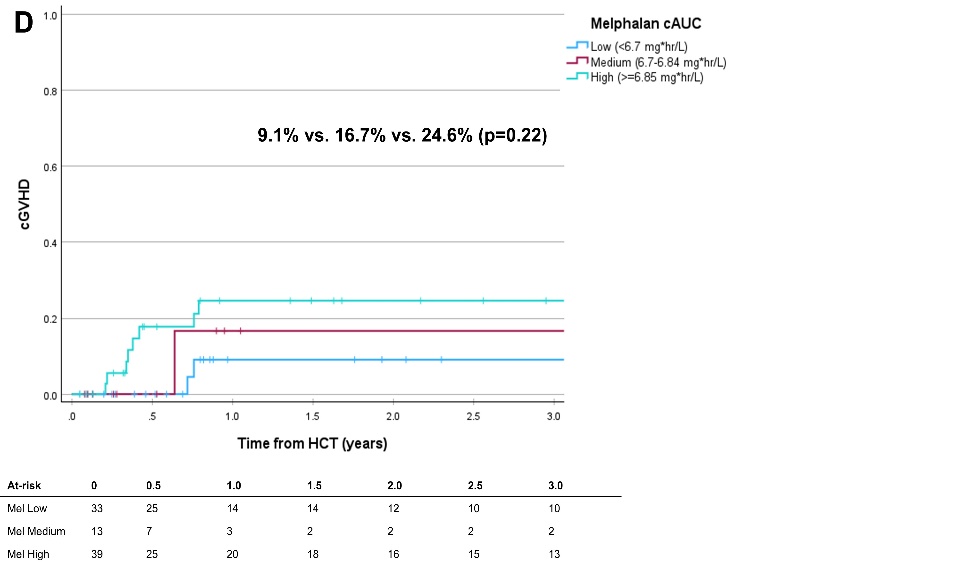
**
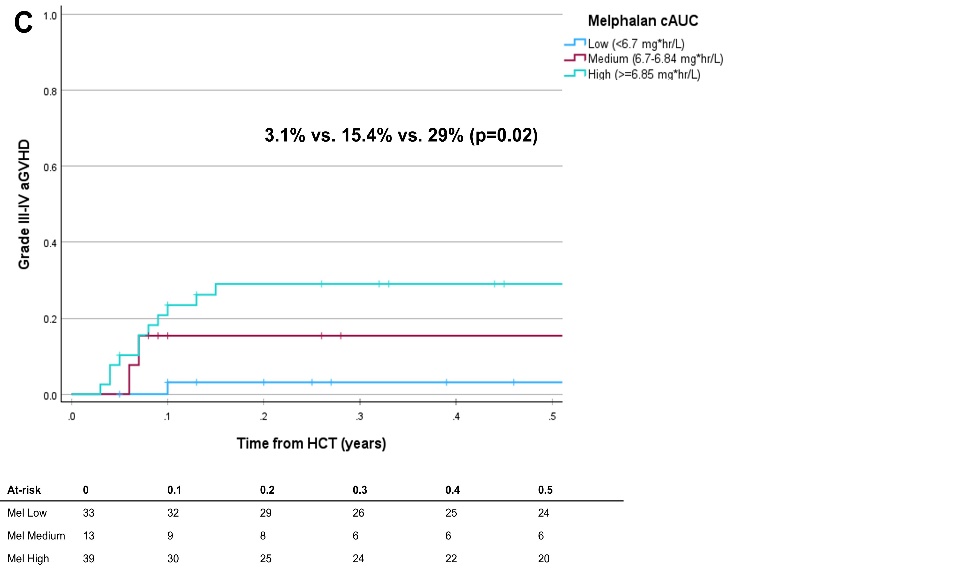
**Grade III-IV Acute GVHD** **Chronic GVHD**


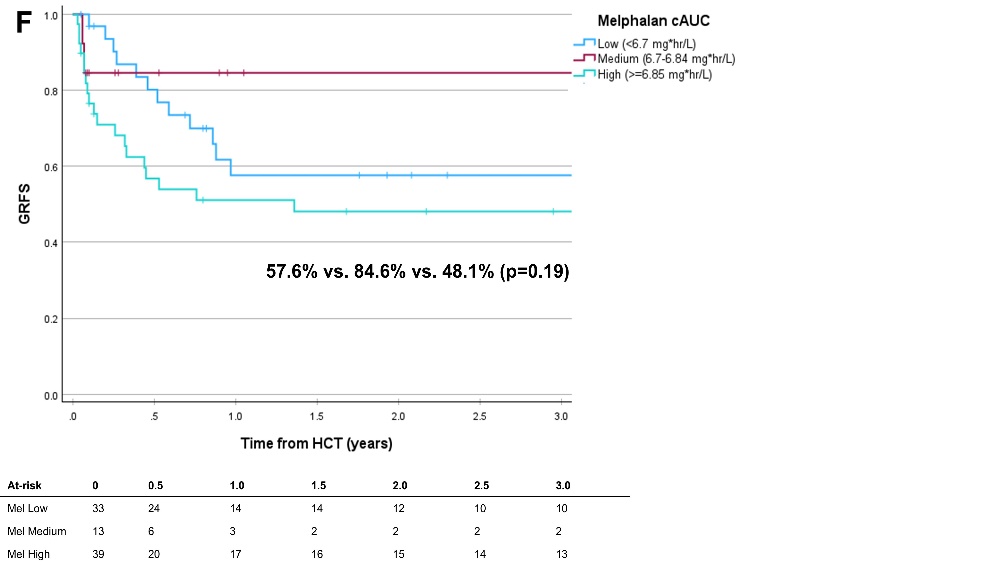

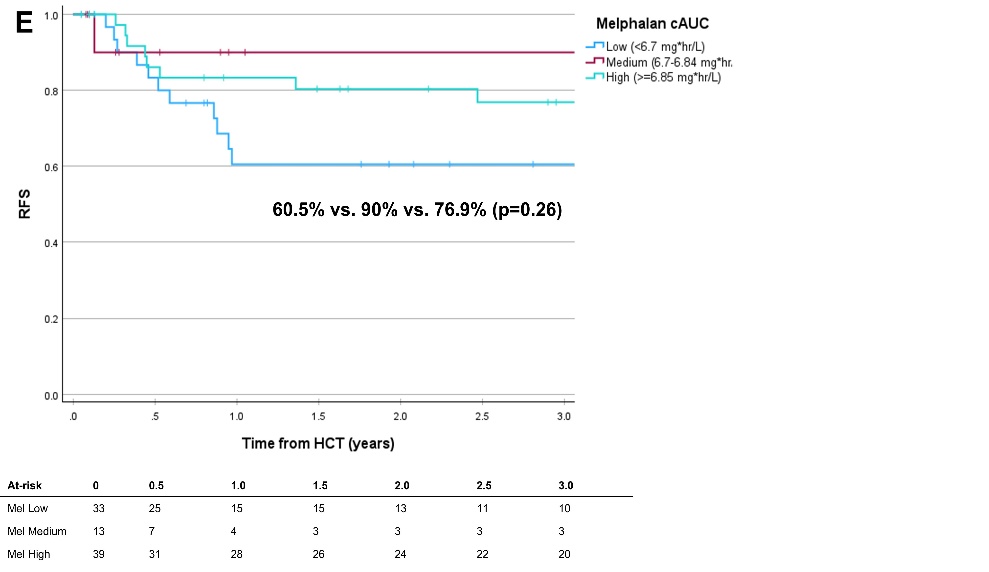
**RFS GRFS**
